# Stability-Based Comparison of Class Discovery Methods for DNA Copy Number Profiles

**DOI:** 10.1371/journal.pone.0081458

**Published:** 2013-12-05

**Authors:** Isabel Brito, Philippe Hupé, Pierre Neuvial, Emmanuel Barillot

**Affiliations:** 1 Institut Curie, Paris, France; 2 INSERM, U900, Paris, France; 3 Mines ParisTech, Fontainebleau, France; 4 CNRS UMR144, Paris, France; 5 Laboratoire Statistique & Génome, Université d′Évry Val d′Essonne, UMR CNRS 8071-USC INRA, Évry, France; Wayne State University, United States of America

## Abstract

**Motivation:**

Array-CGH can be used to determine DNA copy number, imbalances in which are a fundamental factor in the genesis and progression of tumors. The discovery of classes with similar patterns of array-CGH profiles therefore adds to our understanding of cancer and the treatment of patients. Various input data representations for array-CGH, dissimilarity measures between tumor samples and clustering algorithms may be used for this purpose. The choice between procedures is often difficult. An evaluation procedure is therefore required to select the best class discovery method (combination of one input data representation, one dissimilarity measure and one clustering algorithm) for array-CGH. Robustness of the resulting classes is a common requirement, but no stability-based comparison of class discovery methods for array-CGH profiles has ever been reported.

**Results:**

We applied several class discovery methods and evaluated the stability of their solutions, with a modified version of Bertoni's -based test [1]. Our version relaxes the assumption of independency required by original Bertoni's -based test. We conclude that Minimal Regions of alteration (a concept introduced by [2]) for input data representation, sim [3] or agree [4] for dissimilarity measure and the use of average group distance in the clustering algorithm produce the most robust classes of array-CGH profiles.

**Availability:**

The software is available from http://bioinfo.curie.fr/projects/cgh-clustering. It has also been partly integrated into "Visualization and analysis of array-CGH"(VAMP)[5]. The data sets used are publicly available from ACTuDB [6].

## Introduction

Recurrent non random genomic alterations, including changes in DNA copy number in particular, are hallmarks of cancer. The characterization of these imbalances is critical to our understanding of tumorigenesis and cancer progression [7], [8].

Comparative Genomic Hybridization (CGH) is a molecular cytogenetics technique for the efficient characterization of chromosomal gains and losses. Two differently labeled tumoral (test) and healthy (reference) DNA samples are hybridized with normal metaphase chromosome. The relative intensity of the test signal over the reference signal (the signal ratio) reflects the imbalance in copy number between the two samples at a given location (for statistical reasons, ratio are log-transformed and the signal will be termed logratio hereafter). The initial resolution of the technique (about 10 Mbp) improved considerably with the advent of array-based Comparative Genomic Hybridization (array-CGH) in the late 1990s [9], [10]. Array-CGH initially used BAC clone arrays [11] or cDNA arrays [12]. More recently, the use of oligonucleotide arrays [13], [14] or tiling-resolution arrays [15] has further improved the sensitivity and resolution of the technique (typically 20–80 bp for oligonucleotide arrays and about 100 kbp for BAC arrays).

The identification of tumor classes is an important step in cancer research. A class is defined as a family of tumors with similar biological traits and similar clinical features. Class discovery methods have been extensively used for expression data [16] or [17], particularly for tumor classification (e.g. [18]). In this respect, DNA copy number is as crucial as mRNA expression, and biologists and clinicians make use of information concerning genome alterations to investigate tumor biology and to treat patients. For example, chromosome 3 monosomy and chromosome 8q gain is used as an indicator of high metastatic risk in uveal melanoma [19], whereas EGFR amplification is an indication for trastuzumab treatment in breast cancer [20]. However, array-CGH data have specific features differentiating them from expression array data. First, the logratio signals calculated have a small range, which may be discretized into different classes: loss, normal, gain and amplification. Second, neighboring genomic segments are likely to be altered in the same way. Due to these particular features, class discovery for array-CGH data merits a separate analysis, and this constitutes the scope of our work.

Only a few studies dedicated to class discovery for CGH or array-CGH data have been published. [21], [3] and [22] examined chromosomal CGH data whereas [4] explored array-CGH data.

[3] stressed the unusual nature of CGH data and recommended the use of particular dissimilarity measures. They proposed several different dissimilarity measures, the most original of which is sim, which measures the number of contiguous genomic intervals of alterations of the same type overlapping between pairs of samples. [22] presented an algorithm for identifying small sets of important genomic intervals called markers. They showed that markers distinguished effectively between different histological cancer types, thereby improving the quality of clustering.

[4] proposed the WECCA algorithm (weighted clustering of called array-CGH data), a method including a dissimilarity measure and a clustering algorithm devoted to array-CGH data. They defined two dissimilarity measures based on the concepts of agreement (agree) and concordance (conc). Agree is defined as the probability of alterations being identical at the same location in two different samples, under a null model. Conc reflects the similarity in ordering of the types of alteration in two different samples. The clustering algorithm functions as an agglomerative linkage adapted to these two dissimilarity measures and is called total. [4] demonstrated that total linkage is likely to produce tight clusters. Moreover, WECCA produces clusters strongly associated with survival.

Continuing on from these studies, we compared several class discovery methods with a view to identifying the method most appropriate for array-CGH data. We define a class discovery method as the combination of an input data representation, a dissimilarity measure and a clustering algorithm. In many fields, biology and cancer research in particular, it is important for the classes identified to be statistically stable. However, the stability of the classes obtained has never before been estimated for array-CGH data. We therefore tried to determine the best way to obtain stable classes of tumors. Stability is defined as follows: if the class discovery method is applied repeatedly to independent samples and generates similar solutions in each case, then it may be considered statistically stable.

This paper is structured as follows. First, we discuss several possibilities for representing the input data of an array-CGH experiment and we provide a description of array-CGH data preprocessing. Next, we present the dissimilarity measures and clustering algorithms used in this article and the stability-based validation method applied. Then, we show results for several public data sets. Finaly, we present and discuss our results. The mathematical definitions used throughout this article and some tables and figures enclosing results are provided as **Material S1**.

## Input Data Representation Strategies

In an array-CGH experiment, a signal intensity is measured for each probe, for the tumor sample and the reference. The logratio of the signal for the sample to the signal for the reference is calculated and denoted signal logratio. These logratios may be used directly or further processed before their use as input data for classification. It remains unclear which input data representation is optimal for class discovery. Below, we consider several strategies for input data representation for array-CGH classification.

### Strategies using “All probes”

These strategies are straightforward, as they make use of all probes. The input data representation for each probe may be:

- logratio - data are expressed on the base 2 logarithmic scale. This representation is the most common in array-CGH data analysis.
- smoothed logratio - the logratio of the probe is smoothed using its neighbors in the genome. In algorithms such as GLAD [23], the smoothed logratio values are calculated by estimating a piecewise constant function of the raw logratios, using a segmentation procedure. GLAD uses an adaptive weight smoothing algorithm, ensuring that only neighboring probes with similar DNA copy numbers are smoothed together. Several algorithms for the segmentation of array-CGH data have been described (see [24], for a review).
- calls - the data are encoded as discrete and ordinal variables: the calls may be −1 for a probe corresponding to a region of loss, 0 for a normal region, 1 for a region of gain and 2 for a region of amplification.

### Strategies using “Data compression”

In array-CGH data, some probes may be redundant because neighboring genomic segments are likely to be altered in the same way. Data compression strategies involve reducing the number of dimensions so that only a few relevant variables are handled.

#### Statistical compression

The number of dimensions is reduced by Principal Components Analysis (PCA). PCA computes a linear combination of probes that jointly account for most of the variability in the data. PCA is carried out on logratio values and the first components identified constitute the input data representation associated with this strategy.

#### Biological compression

Variable compression is based on the concept of *Minimal Regions* [2]. A *Minimal Region* (MR) is defined as the largest sequence of altered probes (contiguous probes with identical, and not normal, calls) common to a subset of array-CGH profiles, called *support*. Each MR is coded as 1 if the sample belongs to the *support* and as 0 if it does not. Other concepts similar to MR have been proposed, such as *markers* [22], *SIRAC* [25] and *CGHregions* [26].

## Data Pre-Processing

### Data sets

We used five array-CGH data sets publicly available from ACTuDB [6]. Table 1 provides a brief description of each data set, with all datasets identified by the name of the first author.

**Table 1:** Description of array-CGH data sets used in this study.

data set	no. of arrays	no. of probes	platform	tumor tissue
**blaveri** [27]	98	2146	HumArray 2.0	bladder
**gysin** [28]	25	2415	HumArray 2.0	pancreas
**patil** [29]	49	2385	HumArray 1.14	liver
**douglas** [30]	85	3127	BAC/PAC	colon
**veltman** [31]	49	1741	HumArray 1.11	bladder

For all data sets, logratios, smoothed logratios and calls were downloaded from ACTuDB. The sex chromosomes were excluded from the analysis. All data sets presented missing values (between 3 and 13% of the data), which were imputed with the procedure presented in **Material S1** (section **Missing values**). We performed PCA on logratios and retained the principal components jointly accounting for at least 90% of data variability. MR were obtained with VAMP [5], with support ranging from 5 to 50% of the tumors, using increments of 5%.

## Class Discovery Procedures

Mathematical definitions for the items marked  in this section may be found in the **Material S1**. Once the input data representation has been chosen, the class discovery procedure requires the choice of a dissimilarity measure and a clustering algorithm.

### Dissimilarity measures

The objects studied here are tumor samples. As it is not possible to devise a general formula for identifying the best dissimilarity measure for each individual situation, we consider some of the most frequently used methods [32].

We use the general notation *dissimilarity measure* to refer to a distance or a similarity or a dissimilarity. To convert a distance or dissimilarity measure into a similarity measure, or *vice versa*, the value is simply subtracted from the maximum value obtained.

For each input data representation strategy, we calculated different pairwise dissimilarity measures: Euclidean, Manhattan and Pearson correlation. We also calculated the dissimilarity measures proposed by Liu, and by van Wieringen: sim, agree and conc. All three were applied only to calls and biological compression strategies.

sim accounts for the number of contiguous genomic intervals of alterations of the same type overlapping in pairs of samples. In some circumstances, the similarity between one sample and itself may be smaller than that between two different samples (see **Material S1**, section **Dissimilarities**, for an example). To prevent this situation, we made a minor correction: let  be the similarity sim matrix between pairs of samples with generic element , then assign .

The agree measure is defined as the probability of measurements for an arbitrary probe in two different samples being identical and conc is the probability of measurements of an arbitrary probe in two different samples being concordant (i.e. with the same order in terms of magnitude; see [4] for details). These measures are based on the assumption that samples are independent and probes are distributed according to a mixture model.

### Clustering algorithms

Many different clustering algorithms have been described (see [33] for a review).

Hierarchical algorithms are widely used because of their appealing tree representation. Hierarchical agglomerative or bottom-up clustering is a process beginning with the joining of the two most similar objects, with iterative merging of objects or groups of objects until all are included in a single set. By contrast, hierarchical divisive or top-down clustering algorithms begin with the whole set of objects, dividing this set successively in two until each group comprises only one object. Both agglomerative and divisive procedures may be carried out with several linkage methods. In this paper we applied the agglomerative linkages complete, average, weighted and Ward and the divisive linkages diana, tsvq and hybrid.

We also applied partitioning clustering algorithms, which produce flat, non imbricated, clusters. The most common partitioning algorithm is the *k*-means algorithm, which was designed for use with Euclidean distance. We also used a variant, *k*-centroids, which adapts the *k*-means algorithm to other dissimilarity measures. Cluster centroids are defined such that the average dissimilarity of the object of a cluster to all the objects in the cluster is minimal. Finally, we included the algorithm proposed by [4] and called total, which is associated only with agree and conc.

### Evaluation stage

#### Stability

It is difficult to evaluate class discovery solutions, particularly as no class labels are known and so no error rate can be estimated. However, a panoply of criteria for the validation of class discovery solutions has been proposed [34]. External indices assess class discovery solutions according to object labeling, which may be provided by an expert, whereas internal indices evaluate a particular notion of class discovery quality, such as the homogeneity of clusters or the separation of clusters.

We validated class discovery solutions in terms of their stability. Stability is an internal index because it assesses the preservation of class discovery solutions across perturbations of the original data. We compared solutions emerging from two perturbations of the original data, using three coefficients : Simple Matching, Rogers and Tanimoto and Jaccard. These coefficients require a partition to be calculated. To avoid the triky selection of the number of classes, we calculated the coefficients for several partitions.

Several ways of perturbing the data have been proposed. We decided to resample the data by repeatedly drawing overlapping subsets of samples from the same dataset without replacement [35], [36].

#### Assessing the significance of solutions

We assessed the statistical significance of the stability of the structure discovered by the class discovery method, using a modified version of the -based test proposed by [1]. This test was initially designed to determine the number of clusters in a stability framework, but can easily be transposed for class discovery method selection in the same framework, as described below.

A perturbation procedure was applied  times to the data set **X**, building  pairs of subsets of **X**. Let  be a set of  class discovery methods 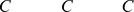.  methods are then applied to the  pairs of subsets and the number  of clusters for each solution is fixed. The similarity of each pair of solutions is then calculated . The  values are the realizations of the random variable .

[1] concluded that  can be used as an index of the reliability of class discovery solutions: if  the solution is stable. The stability of the solution is considered to decrease with increasing distance of  from 1. This result was demonstrated by [1] in the model selection framework, but it also applies *mutatis mutandis* to this context. As we tested a number of methods, we incorporated a multiple testing correction step into the stability analysis.



 may be estimated by its empirical mean , defined as 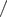.  is then sorted in descending order,  where *p* is a permutation index such that . Class discovery solutions are then ordered from the most to the least stable.

Let us consider the Bernoulli random variable  where  is a fixed threshold,  and *I* is the indicator function. Moreover, consider  Bernoulli random variables  identically distributed with parameter . [1] assumed that the   are independent and identically distributed (i.i.d.).

However, the assumption of independence does not hold in our case since the subsets of **X** may overlap between pairs of subsets. We have therefore extended the Bertoni's -based test in the case the   are dependent and identically distributed with .

Consider , we empirically state that, despite the dependency of the ,  follows a gaussian distribution with parameters  and , for a sufficiently large M. We performed simulations of 100,000 pairs of resamplings which lead to this empirical asymptotic distribution (presented in Figure S1 of **Material S1**, section **Evaluation stage: Assessing the significance of solutions**).

Then, 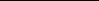 follows an asymptotic standard gaussian distribution. Assuming  i.i.d. for , then under the null hypothesis " for all 
",  may be estimated by the pooled estimate 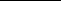. Then, letting 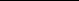 we have under : 


The null hypothesis ": all the  are equal to 
" is tested against the alternative hypothesis ": not all  are equal", with  used as the test statistic. If the null hypothesis is rejected, we exclude the least stable method, according to the sorting of , and repeat the test. *P*-values were adjusted for multiple testing by Bonferroni-Holm correction [37].

This -based test is repeated until no significant difference is detected or until only one class discovery method is left. The set of methods remaining represents the set of stable methods discovered.

All methods are implemented within the R programming language http://www.r-project.org. We used cluster and hybridHclust R packages available from http://www.r-project.org, clusterv and mosclust R packages available from http://homes.dsi.unimi.it/valenti/software.html and WECCA available from http://www.few.vu.nl/wvanwie/software/WECCA/WECCA.html.

## Results

We intensively compared the stability performances of class discovery methods (combinations of an input data representation, a dissimilarity measure and a clustering algorithm). We considered five strategies for input data representation: all versions of All probes (logratio, smoothed logratio and calls), statistical and biological compressions. We considered six dissimilarity measures: Euclidean and Manhattan distances, Pearson correlation and sim, conc and agree similarities. We applied ten clustering algorithms: complete, average, weighted and ward linkages, diana, tsvq, hybrid, *k*-means and *k*-centroids and total. The -based test described was applied iteratively to detect stable class discovery methods.

For all data sets, resampling was performed by establishing  pairs of subsets of each data set. For each subsample, we randomly picked a certain percentage, called resampling rate, of the data set. We considered a spectrum of resampling rates of 50%, 60%, 70%, 80% and 90%.

A dissimilarity measure and a clustering algorithm were applied to each subsample. We considered partitions in  to 10 clusters. For each partition, Simple Matching, Rogers and Tanimoto and Jaccard coefficients were used to compare pairs of solutions from pairs of subsets.

Finally, the -based test was applied iteratively for the detection of stable class discovery solutions for a Bonferroni-Holm-corrected significance level of 5%. The test similarity threshold  was set at 0.85, 0.90, 0.95, 0.97 and 0.99. Moreover, the -based test was computed for each of the 5×3 pairs of thresholds and coefficients in order to assess the robutness of our methodology with respect to the choice of these parameters.

The exhautive results of the 5×3 pairs of thresholds and coefficients are provided in **Material S1** (section **Class discovery methods declared stable**). In what follows, only the results for a resampling rate of 80%, the Jaccard coefficient and 0.97 threshold are reported since the other pairs lead to similar conclusion showing an high robustness of our proposed methodology with respect to the choice of the threshold and the coefficient.

For each data set and each partition, the extensive list of class discovery methods declared stable by the above-described -based test for several test similarity threshold values and several paiwise similarity coefficients is provided in **Material S1**.

**Figure 1:**
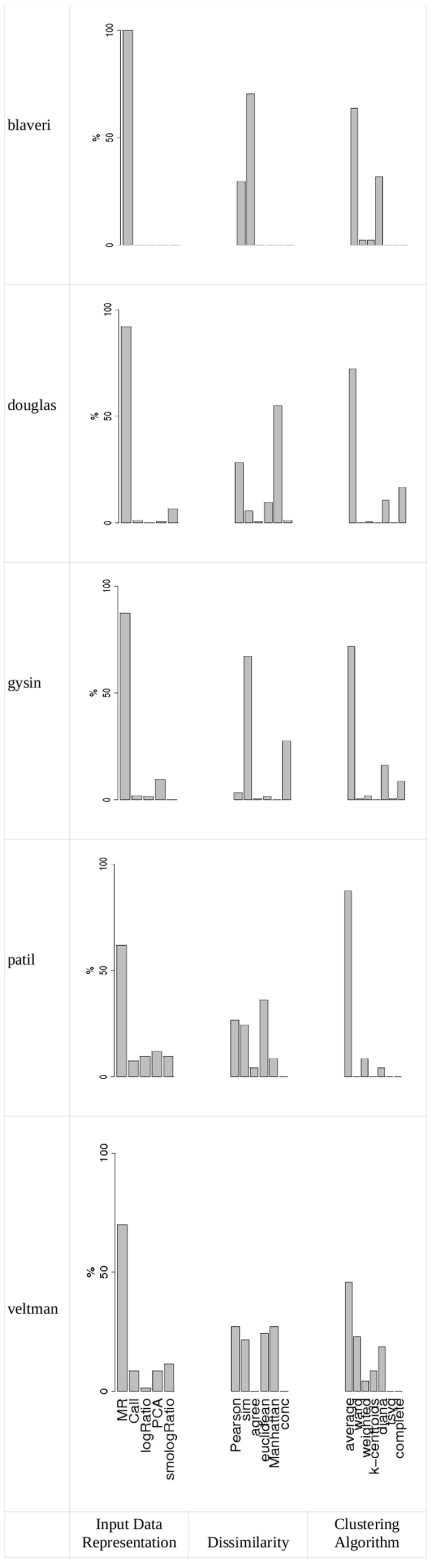
Frequency of input data representation, dissimilarity measure and clustering algorithm among the class discovery methods declared stable for each data set. The parameters used are Jaccard coefficient and 0.97 threshold.

Figure 1 indicates, for each data set, the frequency of each input data representation, each dissimilarity measure and each clustering algorithm in the list of class discovery methods declared stable, all partitions taken together. For all data sets, MR clearly outperformed the other input data representations, and the hierarchical agglomerative linkage average outperformed the other clustering algorithms. The situation is less clear for dissimilarity measures: sim in three cases, agree in one case and Euclidean, Manhattan and Pearson correlation equally outperformed the other dissimilarity measures in one case. The results for each partition are provided in Figures S2 to S7 of **Material S1** and showed that the conclusions remains the same whatever the number of clusters in the partition. However, the rank of the least stable declared class discovery methods may vary as the number of partition increases.

**Figure 2:**
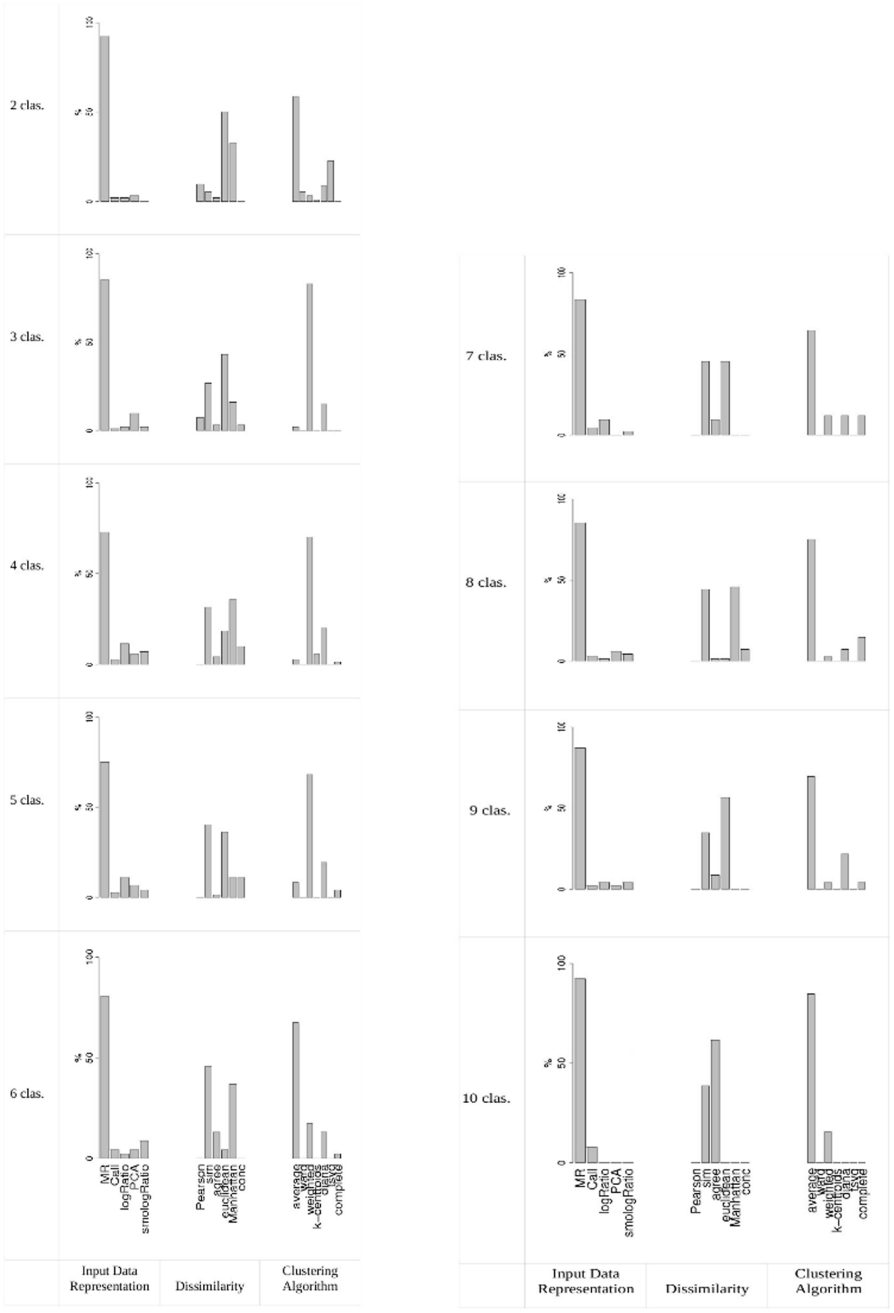
Frequency of input data representation, dissimilarity measure and clustering algorithm among the class discovery methods declared stable for each partition from 2 to 10 clusters. The parameters used are Jaccard coefficient and 0.97 threshold.

We also calculated the frequency of each input data representation, each dissimilarity measure and each clustering algorithm in the class discovery methods declared stable for each partition from 2 to 10, all data sets taken together (see Figure 2). MR and hierarchical average were again identified as the input data representation and clustering algorithm most frequently leading to stable solutions. For dissimilarity measures, Pearson correlation performed well in the case of two clusters and agree performed well with six clusters. For 3, 4, 5, 7, 8, 9 and 10 clusters, sim outperformed the other dissimilarity measures.

The most stable input data representation, dissimilarity measure and clustering algorithm depended little on the data set or number of clusters considered.

**Figure 3:**
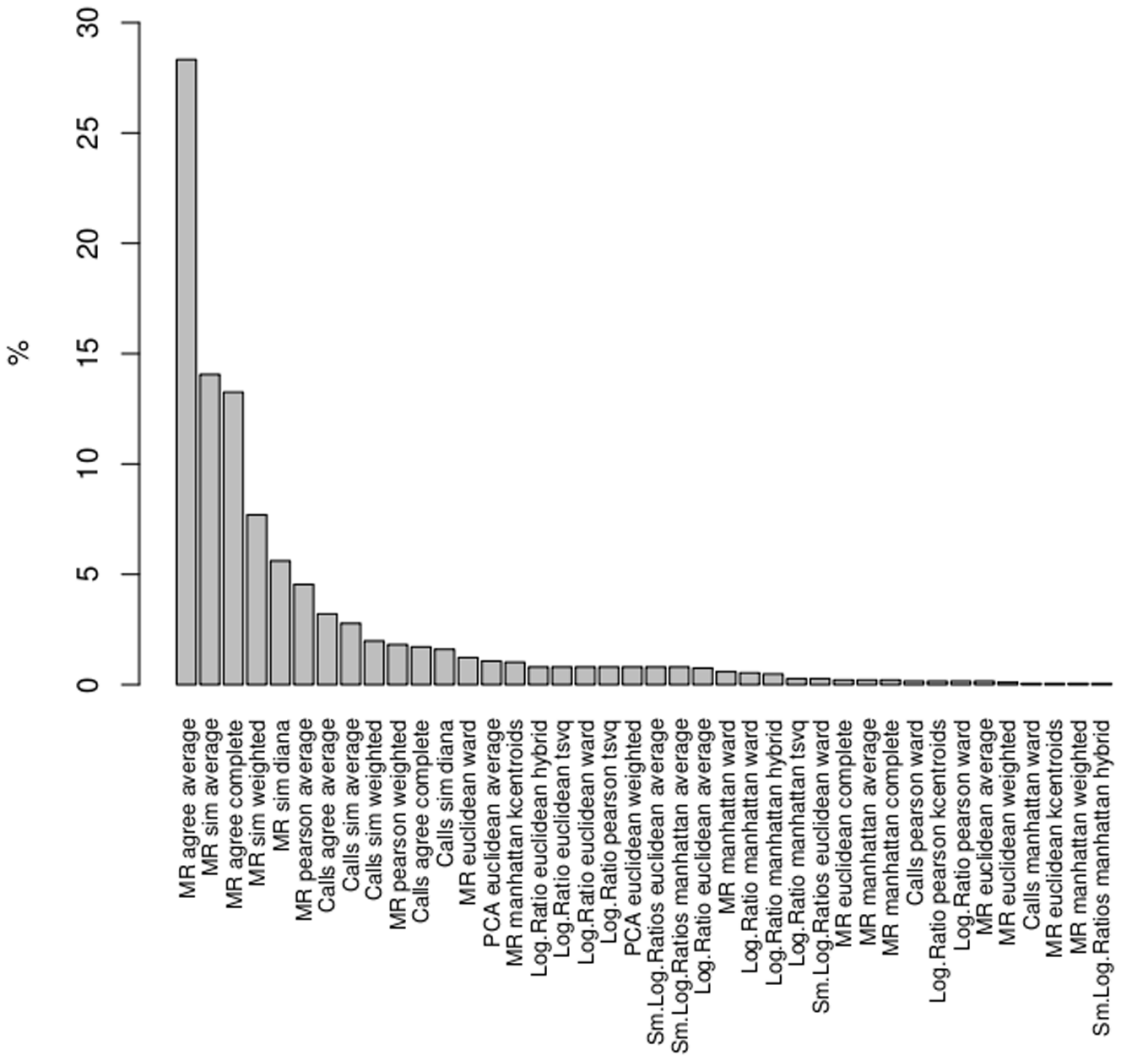
Frequency of class discovery methods declared stable. The parameters used are Jaccard coefficient and 0.97 threshold.

Figure 3 shows the frequency of class discovery methods declared stable over all possible data sets and partitions. The most stable combinations were (MR, agree, average) and (MR, sim, average). By contrast, the hybrid and total algorithms gave no stable solutions.

### Application to Affymetrix SNP arrays

The stability of class discovery methods was evaluated on a public data set with Affymetrix GeneChip Mapping 100K SNP Array Set [38]. Table 2 provides a brief description of the data set.

**Table 2:** Description of data set "zhang"

data set	no. of arrays	no. of probes	platform	tumor tissue
**zhang** [38]	311	58494	Affymetrix 100K Xka	breast

We compared two class discovery methods which were previously declared stable for one (MR, agree, average) and not stable for the other (Calls, euclidean, ward). For this comparison parameters were set essentially as before: resampling with *M* = 100 pairs of subsets of each data set, 80% of the data set was randomly picked for each subsample, *k* = 2 to 6 clusters were considered, Jaccard coefficient was used to evaluate similarity between pairs of solutions, the  -based test similarity *s*  and a Bonferroni-Holm p-value correction.

The results are presented in Table S2 of **Material S1**.

As previously, also for this data set, the combination (MR, agree, average) is declared stable for all possible partitions while the combination (Calls, sim, ward) is declared stable only in one situation and with a lower p-value.

## Discussion and Conclusion

We investigated the application of several input data representations, dissimilarity measures and clustering algorithms for array-CGH data. We compared the resulting class discovery methods in terms of the stability of their solutions.

The two dissimilarities sim and agree appeared to be an efficient choice for array-CGH data in association with MR input data representation. Their superiority can be explained by the fact that they were built taking into account the specificities of array-CGH data. We conclude that the characterization of array-CGH data by MR [2] is a good choice for class discovery purposes, as our experiments demonstrate that stable partitions are generally achieved with this method. As these solutions are reached by reducing the number of data dimensions, the data are characterized in a parsimonious manner. Moreover, the appealing use of MR has already been pointed out by [22], [26] and [25] in classification frameworks. This way, the information is reduced taking into account the redundancy of the data since contiguous probes on the genome are very likely to have the same DNA copy number. This way, the array-CGH profiles are converted into a set of relevant features which leads to more powerful downstream analyses [26], [39].

The use of MR presents other advantages in addition to its parsimony. Firstly, it allows the same weight to be assigned to each alteration, regardless of its size. Indeed, potentially very small alterations, such as amplifications, may be relevant as predictive or prognostic factors. As few probes are found in such small alterations, it may be better to use the alteration as a single entity so that all regions are weighted equally. Secondly, this method facilitates data interpretation because it allows biologists to study a limited number of alterations rather than having to study all the probes to account for differences. Finally, data representation based on MR reduces the amount of data required for class discovery. This feature is particularly useful for high-density array-CGH technologies. To conclude, we recommend the use of hierarchical agglomerative average linkage and sim or agree similarity measures associated with MR for a stable class discovery framework.

## Supporting Information

Material S1Supplementary explanation for the following issues: missing values, dissimilarities, algorithms, partition evaluation, assessing the significance of solutions and lists of class discovery methods declared stable by the -based test for each data set and each partition.(PDF)
