# Supplementary material for: Stability-Based Comparison of Class Discovery Methods for DNA Copy Number Profiles
Source: PLoS One. 2013 Dec 5;8(12):e81458. doi: 10.1371/journal.pone.0081458 (PMC3855312; doi:10.1371/journal.pone.0081458)
Supplement: Material S1 — Supplementary explanation for the following issues: missing values, dissimilarities, algorithms, partition evaluation, assessing the significance of solutions and lists of class discovery methods declared stable by the -based test for each data set and each partition. (PDF) [file pone.0081458.s001.pdf]

Supplementary Material

Missing values

Array-CGH experiments, like most microarray experiments, often generate missing values, due to poor hybridization, high levels of heterogeneity between replicates, image corruption or scratches on the slide.

Several methods have been used to impute missing values for expression data [5]. We propose a novel missing value imputation method more appropriate for array-CGH data. Our method is applied to each sample independently and is based on the *genome metric*. Each probe is assigned a chromosomal position, deduced from its distance in base pairs from the p-telomere.

Missing values are imputed as follows. Let us assume that probe i has a missing value in one sample. Denote by a and b the two probes closest (on the left and right, respectively) to i in the sample.

1. If a and b have the same call, they probably belong to the same genomic region and i naturally belongs to that region, with i given by:
   - The logratio average of a and b as the logratio of i,
   - The interpolation value of the smoothing points of a and b as the *smoothed logratio* of i,
   - The call of a and b as a *call*,
2. If a and b have different calls,
   2.1. If one is normal, preference is given to the alteration and i is given the call, logratio or smoothed logratio of the altered probe.
   2.2. If neither is normal, i is given the call, logratio or smoothed logratio of the probe closest to i.
3. If a has a missing value, then we look for the first probe before a without a missing value and proceed as in 1. or 2. The same procedure is applied if b has a missing value except that we look for the first probe after b.

Dissimilarities

Let $\mathbf{X}$ be the CGH-array data set matrix with each element representing the call of one probe on one sample. The similarity between samples j and l for $j, l = 1, \ldots, p$ is $s(j, l)$ defined as

sim

$s(j, l) =$ number of overlapping segments (ovs) between j and l

In some circumstances, the similarity between one sample and itself may be be smaller than between two different samples. For exemple, for the two samples j and l below, $s(j, l) = 2$ and $s(j, j) = 1$.

Probes	1	2	3	4	5	6	7	8	9
j	0	0	1	1	1	1	1	0	0
l	0	1	1	-1	-1	1	1	0	0
ovs			—			—	—		

Probes	1	2	3	4	5	6	7	8	9
j	0	0	1	1	1	1	1	0	0
j	0	0	1	1	1	1	1	0	0
ovs			—	—	—	—			

To prevent this situation, we made the following correction. If $\exists\ s_{j,l} > s_{j,j}$, then assign $s_{j,j} = max\ s_{j,l}$.

Algorithms

Let D be the matrix of dissimilarities between pairs of ojects with generic element d_{jl} for $j,\ l = 1,\ \ldots,\ p$. If similarities are mesured instead, they are converted to dissimilarities as all clustering algorithms here used need dissimilarities. Let G_k and G_u for $k,\ u = 1,\ \ldots,\ K$ be two clusters obtained by some clustering algorithm.

Hierarchical agglomerative clustering is a process beginning with the joining of the two most similar objects, with iterative merging of objects or groups of objects until all are included in a single set. Several linkage methods or ways to construct a distance between groups of objects exist. Some of them are described below. Let T be the matrix of distances between pairs of clusters with generic element $t(G_k, G_u)$ and let n_{G_k} be the number of objects in cluster G_k.

<u>`complete` linkage</u>

$$t(G_k, G_u) = max_{j \in G_k,\ l \in G_u} d(j, l)$$

<u>`average` linkage</u>

$$t(G_k, G_u) = \frac{1}{n_{G_k} n_{G_u}} \sum_{j \in G_k,\ l \in G_u} d(j, l)$$

<u>`weigthed` linkage</u>
Is a variant of `average` linkage. `Average` linkage assigns to all pairs of objects the same weight. By contrast, `weigthed` linkage assigns equal weight to each group. It does not correspond to any inherent definition of distance between clusters but it can be expressed by the following updating equation (see [1]). At each fusion of clusters G_{k_1} and G_{k_2}, forming some new cluster G_k, the distances between clusters are updated by

$$t(G_k, G_u) = \frac{1}{2} t(G_{k_1}, G_u) + \frac{1}{2} t(G_{k_2}, G_u)$$

<u>`ward`'s linkage</u>

$$t(G_k, G_u) = \sqrt{\frac{2 n_{G_k} n_{G_u}}{n_{G_k} + n_{G_u}}}\ d(\bar{x}(G_k) - \bar{x}(G_u))$$

where $\bar{x}(G_k)$ and $\bar{x}(G_u)$ represent the centroids of clusters G_k and G_u.

Hierarchical divisive methods begin with the whole set of objects, dividing this set successively in two until each group comprises only one object. However, as it is not possible to consider all possible two-groups divisions for computation time reason, heuristic algorithms are used. Among them, we have used the following divisive clustering algorithms:

<u>`diana`</u>
Uses the algorithm described by [2] which is based on average distances inside clusters $\frac{1}{n_{G_k}} \sum_{j,l \in G_k} d(j, l)$.

<u>`tsvq`</u>
Tree Structured Vector Quantization (`tsvq`) was proposed by [4] and performs at each division a k-means with k=2.

<u>`hybrid`</u>
`Hybrid` clustering works as `tsvq` but avoid certain clusters to be split depending on mutual notion defined by [3].

Partition evaluation

Let K_1 and K_2 be two different partitions of the p samples, we define the number of pairs of samples (nps) which are in the following conditions as

N_{11}	nps that are placed in the same cluster in K_1 and also in K_2
N_{10}	nps that are placed in the same cluster in K_1 but not in K_2
N_{01}	nps that are placed in different clusters in K_1 but in the same cluster in K_2
N_{00}	nps that are placed in different clusters in K_1 and in different clusters in K_2

The following coefficients (see [6]) evaluate how similar are partitions K_1 and K_2.

Simple Matching coefficient

$$SM(K_1, K_2) = \frac{N_{11} + N_{00}}{p}$$

Rogers and Tanimoto coefficient

$$RT(K_1, K_2) = \frac{N_{11} + N_{00}}{N_{11} + N_{00} + 2N_{10} + 2N_{01}}$$

Jaccard coefficient

$$JC(K_1, K_2) = \frac{N_{11}}{N_{01} + N_{10} + N_{11}}$$

Evaluation stage: Assessing the significance of solutions

We provide empirical evidence for the asymptotic standard normal distribution of Z_r.

In this intention, we consider the class discovery method (MR, sim, average) on the dataset blaveri. Other parameters are resampling with $M = 100$ pairs of subsets of data set, randomly resampling rate of 80%, $k = 2$ clusters, Jaccard coefficient to compute similarities between pairs of solutions, test similarity threshold s_0 of 0.97.

This framework was replayed 1,000 times and each time we computed the realisation of X_r, which is the sum of $I(S_{rm} > s_0)$. The parameter θ_r was estimated as $1/M$ times the empirical mean of these 1,000 realisations.

The histogram of the distribution of Z_r compared to standard normal distribution (in red) is portrayed in Figure S1 (left). The same comparison is performed with the Normal Q-Q Plot and represented in Figure S1 (right).

Based on this empirical evidence, we can reasonably state that Z_r approximately follows a gaussian distribution with parameters $M\theta_r$ and $M\theta_r(1 - \theta_r)$, for a sufficiently large M. We then define,

$$Y = \sum_{r=1}^{R} \frac{(X_r - M\hat{\theta})^2}{M\hat{\theta}(1 - \hat{\theta})} \overset{M\to\infty}{\sim} \chi^2(R - 1)$$

where $\hat{\theta}$ is defined as the pooled estimate $\frac{\sum_{r=1}^{R} X_r}{RM}$.

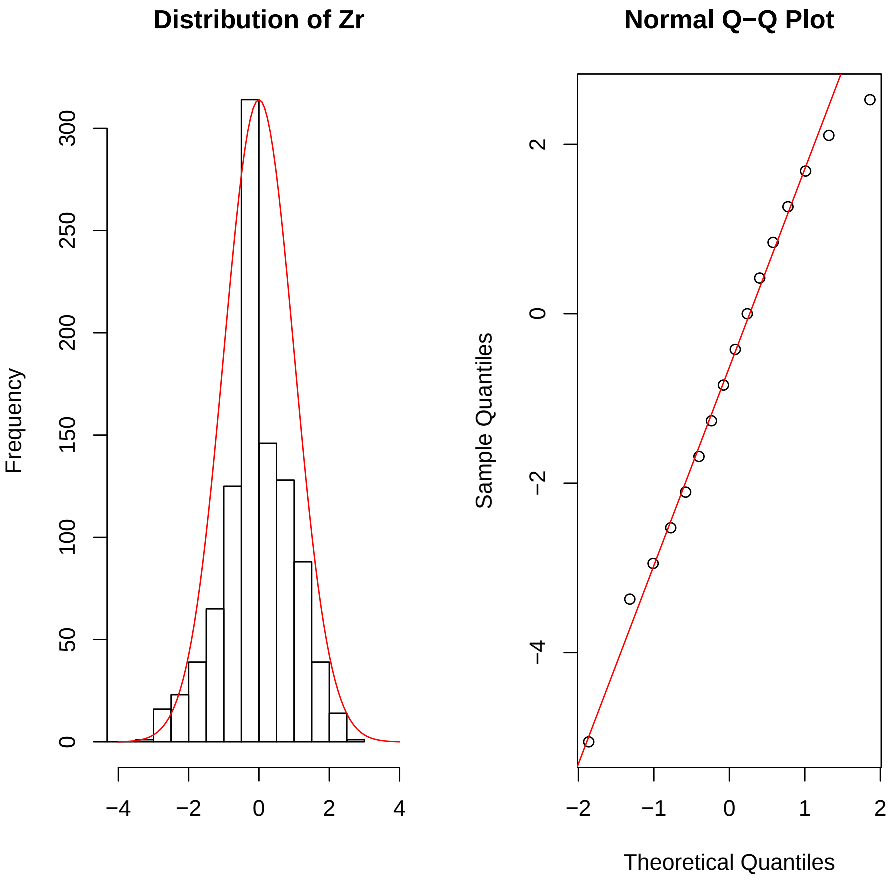

Figure S 1. (left) Distribution of Z_r and standard normal distribution (in red). (right) Normal Q-Q Plot for the distribution of Z_r

Class discovery methods declared stable by the χ^2-based test for each data set and each partition

The p-values provided in Tables 1 to .. are the adjusted p-values by Bonferroni-Holm method. They correspond to the first test of the iteration process not rejecting the "H_0" null hypothesis. We remind that in the context of the χ^2-based test, "H_0: all the θ_r are equal to θ" is tested against the "H_1: not all θ_k are equal", with Y used as the test statistic. If the null hypothesis is rejected, we exclude the least stable method and repeat the test.

This test was performed for each of

- data sets: veltman, douglas, gysin, blaveri, patil,
- partitions 2 to 10 clusters,
- resampling rate of 80%,
  - similarity coefficients: Jaccard, Single Matching, Rogers-Tanimoto,
  - similarity threshold s^0 of 0.85, 0.90, 0.95, 0.97, 0.99,
- resampling rates of 50%, 60%, 70% and 90%,
  - similarity coefficient: Jaccard,
  - similarity threshold s^0 of 0.97.

set = veltman		
resampling rate=80%, similarity coefficient=Jaccard, $s^0 = 0.85$		
**nb.clusters**		**p-value**
2	MR pearson average	0.0603
	MR pearson ward	
	MR pearson weighted	
	MR pearson kcentroids	
	MR euclidean diana	
	MR manhattan diana	
3	MR pearson average	0.5352
	MR pearson weighted	
4	MR euclidean average	0.0922
	MR manhattan average	
	RATIO euclidean average	
	STATUS euclidean average	
	RATIO euclidean diana	
	SMOOTH euclidean average	
	RATIO manhattan diana	
	RATIO manhattan average	
	SMOOTH euclidean diana	
	SMOOTH manhattan average	
	MR euclidean diana	
	MR manhattan diana	
	SMOOTH manhattan diana	
	STATUS euclidean diana	
	STATUS euclidean weighted	
	MR manhattan kcentroids	
5	SMOOTH manhattan average	1
	STATUS euclidean average	
	MR manhattan diana	
	SMOOTH manhattan diana	
	RATIO manhattan average	
	MR euclidean diana	
	MR euclidean average	
	MR manhattan average	
	STATUS manhattan average	
6	MR euclidean average	0.1394
	MR manhattan average	
	STATUS manhattan weighted	
7	MR euclidean average	1
8	SMOOTH euclidean diana	1
9	MR sim average	1
	MR sim weighted	
	ACPrat euclidean diana	
	SMOOTH euclidean average	
	RATIO euclidean diana	
10	MR sim average	1
	MR sim weighted	

Table S 1. Stable class discovery methods and associated p-value for data set **veltman**, partitions from 2 to 10 clusters, resampling rate of 80%, similarity coefficient Jaccard and similarity threshold $s^0 = 0.85$.

set = veltman		
resampling rate=80%, similarity coefficient=Jaccard, $s^0 = 0.90$		
**nb.clusters**		**p-value**
2	MR pearson average	0.0603
	MR pearson ward	
	MR pearson weighted	
	MR pearson kcentroids	
	MR euclidean diana	
	MR manhattan diana	
3	MR pearson average	0.5352
	MR pearson weighted	
4	MR euclidean average	0.0922
	MR manhattan average	
	RATIO euclidean average	
	STATUS euclidean average	
	RATIO euclidean diana	
	SMOOTH euclidean average	
	RATIO manhattan diana	
	RATIO manhattan average	
	SMOOTH euclidean diana	
	SMOOTH manhattan average	
	MR euclidean diana	
	MR manhattan diana	
	SMOOTH manhattan diana	
	STATUS euclidean diana	
	STATUS euclidean weighted	
	MR manhattan kcentroids	
5	SMOOTH manhattan average	1
	STATUS euclidean average	
	MR manhattan diana	
	SMOOTH manhattan diana	
	RATIO manhattan average	
	MR euclidean diana	
	MR euclidean average	
	MR manhattan average	
	STATUS manhattan average	
6	MR euclidean average	0.1394
	MR manhattan average	
	STATUS manhattan weighted	
7	MR euclidean average	1
8	SMOOTH euclidean diana	1
9	MR sim average	1
	MR sim weighted	
	ACPrat euclidean diana	
	SMOOTH euclidean average	
	RATIO euclidean diana	
10	MR sim average	1
	MR sim weighted	

Table S 2. Stable class discovery methods and associated p-value for data set **veltman**, partitions from 2 to 10 clusters, resampling rate of 80%, similarity coefficient Jaccard and similarity threshold $s^0 = 0.90$.

set = veltman		
resampling rate=80%, similarity coefficient=Jaccard, $s^0 = 0.95$		
**nb.clusters**		**p-value**
2	MR pearson average	0.0603
	MR pearson ward	
	MR pearson weighted	
	MR pearson kcentroids	
	MR euclidean diana	
	MR manhattan diana	
3	MR pearson average	0.5352
	MR pearson weighted	
4	MR euclidean average	0.0922
	MR manhattan average	
	RATIO euclidean average	
	STATUS euclidean average	
	RATIO euclidean diana	
	SMOOTH euclidean average	
	RATIO manhattan diana	
	RATIO manhattan average	
	SMOOTH euclidean diana	
	SMOOTH manhattan average	
	MR euclidean diana	
	MR manhattan diana	
	SMOOTH manhattan diana	
	STATUS euclidean diana	
	STATUS euclidean weighted	
	MR manhattan kcentroids	
5	SMOOTH manhattan average	1
	STATUS euclidean average	
	MR manhattan diana	
	SMOOTH manhattan diana	
	RATIO manhattan average	
	MR euclidean diana	
	MR euclidean average	
	MR manhattan average	
	STATUS manhattan average	
6	MR euclidean average	0.1394
	MR manhattan average	
	STATUS manhattan weighted	
7	MR euclidean average	1
8	SMOOTH euclidean diana	1
9	MR sim average	1
	MR sim weighted	
	ACPrat euclidean diana	
	SMOOTH euclidean average	
	RATIO euclidean diana	
10	MR sim average	1
	MR sim weighted	

Table S 3. Stable class discovery methods and associated p-value for data set **veltman**, partitions from 2 to 10 clusters, resampling rate of 80%, similarity coefficient Jaccard and similarity threshold $s^0 = 0.95$.

set = veltman		
resampling rate=80%, similarity coefficient=Jaccard, $s^0 = 0.97$		
**nb.clusters**		**p-value**
2	MR pearson average	0.0603
	MR pearson ward	
	MR pearson weighted	
	MR pearson kcentroids	
	MR euclidean diana	
	MR manhattan diana	
3	MR pearson average	0.5352
	MR pearson weighted	
4	MR euclidean average	0.0922
	MR manhattan average	
	RATIO euclidean average	
	STATUS euclidean average	
	RATIO euclidean diana	
	SMOOTH euclidean average	
	RATIO manhattan diana	
	RATIO manhattan average	
	SMOOTH euclidean diana	
	SMOOTH manhattan average	
	MR euclidean diana	
	MR manhattan diana	
	SMOOTH manhattan diana	
	STATUS euclidean diana	
	STATUS euclidean weighted	
	MR manhattan kcentroids	
5	SMOOTH manhattan average	1
	STATUS euclidean average	
	MR manhattan diana	
	SMOOTH manhattan diana	
	RATIO manhattan average	
	MR euclidean diana	
	MR euclidean average	
	MR manhattan average	
	STATUS manhattan average	
6	MR euclidean average	0.1394
	MR manhattan average	
	STATUS manhattan weighted	
7	MR euclidean average	1
8	SMOOTH euclidean diana	1
9	MR sim average	1
	MR sim weighted	
	ACPrat euclidean diana	
	SMOOTH euclidean average	
	RATIO euclidean diana	
10	MR sim average	1
	MR sim weighted	

Table S 4. Stable class discovery methods and associated p-value for data set **veltman**, partitions from 2 to 10 clusters, resampling rate of 80%, similarity coefficient Jaccard and similarity threshold $s^0 = 0.97$.

set = veltman		
resampling rate=80%, similarity coefficient=Jaccard, $s^0 = 0.99$		
**nb.clusters**		**p-value**
2	MR pearson average	0.0603
	MR pearson ward	
	MR pearson weighted	
	MR pearson kcentroids	
	MR euclidean diana	
	MR manhattan diana	
3	MR pearson average	0.5352
	MR pearson weighted	
4	MR euclidean average	0.0922
	MR manhattan average	
	RATIO euclidean average	
	STATUS euclidean average	
	RATIO euclidean diana	
	SMOOTH euclidean average	
	RATIO manhattan diana	
	RATIO manhattan average	
	SMOOTH euclidean diana	
	SMOOTH manhattan average	
	MR euclidean diana	
	MR manhattan diana	
	SMOOTH manhattan diana	
	STATUS euclidean diana	
	STATUS euclidean weighted	
	MR manhattan kcentroids	
5	SMOOTH manhattan average	1
	STATUS euclidean average	
	MR manhattan diana	
	SMOOTH manhattan diana	
	RATIO manhattan average	
	MR euclidean diana	
	MR euclidean average	
	MR manhattan average	
	STATUS manhattan average	
6	MR euclidean average	0.1394
	MR manhattan average	
	STATUS manhattan weighted	
7	MR euclidean average	1
8	SMOOTH euclidean diana	1
9	MR sim average	1
	MR sim weighted	
	ACPrat euclidean diana	
	SMOOTH euclidean average	
	RATIO euclidean diana	
10	MR sim average	1
	MR sim weighted	

Table S 5. Stable class discovery methods and associated p-value for data set **veltman**, partitions from 2 to 10 clusters, resampling rate of 80%, similarity coefficient Jaccard and similarity threshold $s^0 = 0.99$.

set = veltman		
resampling rate=80%, similarity coefficient=Simple Matching, $s^0 = 0.85$		
**nb.clusters**		**p-value**
2	MR pearson average	0.0603
	MR pearson ward	
	MR pearson weighted	
	MR pearson kcentroids	
	MR euclidean diana	
	MR manhattan diana	
3	MR pearson average	0.5352
	MR pearson weighted	
4	MR euclidean average	0.0922
	MR manhattan average	
	RATIO euclidean average	
	STATUS euclidean average	
	RATIO euclidean diana	
	SMOOTH euclidean average	
	RATIO manhattan diana	
	RATIO manhattan average	
	SMOOTH euclidean diana	
	SMOOTH manhattan average	
	MR euclidean diana	
	MR manhattan diana	
	SMOOTH manhattan diana	
	STATUS euclidean diana	
	STATUS euclidean weighted	
	MR manhattan kcentroids	
5	SMOOTH manhattan average	1
	STATUS euclidean average	
	MR manhattan diana	
	SMOOTH manhattan diana	
	RATIO manhattan average	
	MR euclidean diana	
	MR euclidean average	
	MR manhattan average	
	STATUS manhattan average	
6	MR euclidean average	0.1394
	MR manhattan average	
	STATUS manhattan weighted	
7	MR euclidean average	1
8	SMOOTH euclidean diana	1
9	MR sim average	1
	MR sim weighted	
	ACPrat euclidean diana	
	SMOOTH euclidean average	
	RATIO euclidean diana	
10	MR sim average	1
	MR sim weighted	

Table S 6. Stable class discovery methods and associated p-value for data set **veltman**, partitions from 2 to 10 clusters, resampling rate of 80%, similarity coefficient Simple Matching and similarity threshold $s^0 = 0.85$.

**set = veltman**		
resampling rate=80%, similarity coefficient=Simple Matching, $s^0 = 0.90$		
**nb.clusters**		**p-value**
2	MR pearson average	0.0603
	MR pearson ward	
	MR pearson weighted	
	MR pearson kcentroids	
	MR euclidean diana	
	MR manhattan diana	
3	MR pearson average	0.5352
	MR pearson weighted	
4	MR euclidean average	0.0922
	MR manhattan average	
	RATIO euclidean average	
	STATUS euclidean average	
	RATIO euclidean diana	
	SMOOTH euclidean average	
	RATIO manhattan diana	
	RATIO manhattan average	
	SMOOTH euclidean diana	
	SMOOTH manhattan average	
	MR euclidean diana	
	MR manhattan diana	
	SMOOTH manhattan diana	
	STATUS euclidean diana	
	STATUS euclidean weighted	
	MR manhattan kcentroids	
5	SMOOTH manhattan average	1
	STATUS euclidean average	
	MR manhattan diana	
	SMOOTH manhattan diana	
	RATIO manhattan average	
	MR euclidean diana	
	MR euclidean average	
	MR manhattan average	
	STATUS manhattan average	
6	MR euclidean average	0.1394
	MR manhattan average	
	STATUS manhattan weighted	
7	MR euclidean average	1
8	SMOOTH euclidean diana	1
9	MR sim average	1
	MR sim weighted	
	ACPrat euclidean diana	
	SMOOTH euclidean average	
	RATIO euclidean diana	
10	MR sim average	1
	MR sim weighted	

Table S 7. Stable class discovery methods and associated p-value for data set **veltman**, partitions from 2 to 10 clusters, resampling rate of 80%, similarity coefficient Simple Matching and similarity threshold $s^0 = 0.90$.

set = veltman		
resampling rate=80%, similarity coefficient=Simple Matching, $s^0 = 0.95$		
**nb.clusters**		**p-value**
2	MR pearson average	0.0603
	MR pearson ward	
	MR pearson weighted	
	MR pearson kcentroids	
	MR euclidean diana	
	MR manhattan diana	
3	MR pearson average	0.5352
	MR pearson weighted	
4	MR euclidean average	0.0922
	MR manhattan average	
	RATIO euclidean average	
	STATUS euclidean average	
	RATIO euclidean diana	
	SMOOTH euclidean average	
	RATIO manhattan diana	
	RATIO manhattan average	
	SMOOTH euclidean diana	
	SMOOTH manhattan average	
	MR euclidean diana	
	MR manhattan diana	
	SMOOTH manhattan diana	
	STATUS euclidean diana	
	STATUS euclidean weighted	
	MR manhattan kcentroids	
5	SMOOTH manhattan average	1
	STATUS euclidean average	
	MR manhattan diana	
	SMOOTH manhattan diana	
	RATIO manhattan average	
	MR euclidean diana	
	MR euclidean average	
	MR manhattan average	
	STATUS manhattan average	
6	MR euclidean average	0.1394
	MR manhattan average	
	STATUS manhattan weighted	
7	MR euclidean average	1
8	SMOOTH euclidean diana	1
9	MR sim average	1
	MR sim weighted	
	ACPrat euclidean diana	
	SMOOTH euclidean average	
	RATIO euclidean diana	
10	MR sim average	1
	MR sim weighted	

Table S 8. Stable class discovery methods and associated p-value for data set **veltman**, partitions from 2 to 10 clusters, resampling rate of 80%, similarity coefficient Simple Matching and similarity threshold $s^0 = 0.95$.

set = veltman		
resampling rate=80%, similarity coefficient=Simple Matching, $s^0 = 0.97$		
**nb.clusters**		**p-value**
2	MR pearson average	0.0603
	MR pearson ward	
	MR pearson weighted	
	MR pearson kcentroids	
	MR euclidean diana	
	MR manhattan diana	
3	MR pearson average	0.5352
	MR pearson weighted	
4	MR euclidean average	0.0922
	MR manhattan average	
	RATIO euclidean average	
	STATUS euclidean average	
	RATIO euclidean diana	
	SMOOTH euclidean average	
	RATIO manhattan diana	
	RATIO manhattan average	
	SMOOTH euclidean diana	
	SMOOTH manhattan average	
	MR euclidean diana	
	MR manhattan diana	
	SMOOTH manhattan diana	
	STATUS euclidean diana	
	STATUS euclidean weighted	
	MR manhattan kcentroids	
5	SMOOTH manhattan average	1
	STATUS euclidean average	
	MR manhattan diana	
	SMOOTH manhattan diana	
	RATIO manhattan average	
	MR euclidean diana	
	MR euclidean average	
	MR manhattan average	
	STATUS manhattan average	
6	MR euclidean average	0.1394
	MR manhattan average	
	STATUS manhattan weighted	
7	MR euclidean average	1
8	SMOOTH euclidean diana	1
9	MR sim average	1
	MR sim weighted	
	ACPrat euclidean diana	
	SMOOTH euclidean average	
	RATIO euclidean diana	
10	MR sim average	1
	MR sim weighted	

Table S 9. Stable class discovery methods and associated p-value for data set **veltman**, partitions from 2 to 10 clusters, resampling rate of 80%, similarity coefficient Simple Matching and similarity threshold $s^0 = 0.97$.

set = veltman		
resampling rate=80%, similarity coefficient=Simple Matching, $s^0 = 0.99$		
**nb.clusters**		**p-value**
2	MR pearson average	0.0603
	MR pearson ward	
	MR pearson weighted	
	MR pearson kcentroids	
	MR euclidean diana	
	MR manhattan diana	
3	MR pearson average	0.5352
	MR pearson weighted	
4	MR euclidean average	0.0922
	MR manhattan average	
	RATIO euclidean average	
	STATUS euclidean average	
	RATIO euclidean diana	
	SMOOTH euclidean average	
	RATIO manhattan diana	
	RATIO manhattan average	
	SMOOTH euclidean diana	
	SMOOTH manhattan average	
	MR euclidean diana	
	MR manhattan diana	
	SMOOTH manhattan diana	
	STATUS euclidean diana	
	STATUS euclidean weighted	
	MR manhattan kcentroids	
5	SMOOTH manhattan average	1
	STATUS euclidean average	
	MR manhattan diana	
	SMOOTH manhattan diana	
	RATIO manhattan average	
	MR euclidean diana	
	MR euclidean average	
	MR manhattan average	
	STATUS manhattan average	
6	MR euclidean average	0.1394
	MR manhattan average	
	STATUS manhattan weighted	
7	MR euclidean average	1
8	SMOOTH euclidean diana	1
9	MR sim average	1
	MR sim weighted	
	ACPrat euclidean diana	
	SMOOTH euclidean average	
	RATIO euclidean diana	
10	MR sim average	1
	MR sim weighted	

Table S 10. Stable class discovery methods and associated p-value for data set **veltman**, partitions from 2 to 10 clusters, resampling rate of 80%, similarity coefficient Simple Matching and similarity threshold $s^0 = 0.99$.

**set = veltman**		
resampling rate=80%, similarity coefficient=Rogers-Tanimoto, $s^0 = 0.85$		
**nb.clusters**		**p-value**
2	MR pearson average	0.0603
	MR pearson ward	
	MR pearson weighted	
	MR pearson kcentroids	
	MR euclidean diana	
	MR manhattan diana	
3	MR pearson average	0.5352
	MR pearson weighted	
4	MR euclidean average	0.0922
	MR manhattan average	
	RATIO euclidean average	
	STATUS euclidean average	
	RATIO euclidean diana	
	SMOOTH euclidean average	
	RATIO manhattan diana	
	RATIO manhattan average	
	SMOOTH euclidean diana	
	SMOOTH manhattan average	
	MR euclidean diana	
	MR manhattan diana	
	SMOOTH manhattan diana	
	STATUS euclidean diana	
	STATUS euclidean weighted	
	MR manhattan kcentroids	
5	SMOOTH manhattan average	1
	STATUS euclidean average	
	MR manhattan diana	
	SMOOTH manhattan diana	
	RATIO manhattan average	
	MR euclidean diana	
	MR euclidean average	
	MR manhattan average	
	STATUS manhattan average	
6	MR euclidean average	0.1394
	MR manhattan average	
	STATUS manhattan weighted	
7	MR euclidean average	1
8	SMOOTH euclidean diana	1
9	MR sim average	1
	MR sim weighted	
	ACPrat euclidean diana	
	SMOOTH euclidean average	
	RATIO euclidean diana	
10	MR sim average	1
	MR sim weighted	

Table S 11. Stable class discovery methods and associated p-value for data set **veltman**, partitions from 2 to 10 clusters, resampling rate of 80%, similarity coefficient Rogers-Tanimoto and similarity threshold $s^0 = 0.85$.

set = veltman		
resampling rate=80%, similarity coefficient=Rogers-Tanimoto, $s^0 = 0.90$		
**nb.clusters**		**p-value**
2	MR pearson average	0.0603
	MR pearson ward	
	MR pearson weighted	
	MR pearson kcentroids	
	MR euclidean diana	
	MR manhattan diana	
3	MR pearson average	0.5352
	MR pearson weighted	
4	MR euclidean average	0.0922
	MR manhattan average	
	RATIO euclidean average	
	STATUS euclidean average	
	RATIO euclidean diana	
	SMOOTH euclidean average	
	RATIO manhattan diana	
	RATIO manhattan average	
	SMOOTH euclidean diana	
	SMOOTH manhattan average	
	MR euclidean diana	
	MR manhattan diana	
	SMOOTH manhattan diana	
	STATUS euclidean diana	
	STATUS euclidean weighted	
	MR manhattan kcentroids	
5	SMOOTH manhattan average	1
	STATUS euclidean average	
	MR manhattan diana	
	SMOOTH manhattan diana	
	RATIO manhattan average	
	MR euclidean diana	
	MR euclidean average	
	MR manhattan average	
	STATUS manhattan average	
6	MR euclidean average	0.1394
	MR manhattan average	
	STATUS manhattan weighted	
7	MR euclidean average	1
8	SMOOTH euclidean diana	1
9	MR sim average	1
	MR sim weighted	
	ACPrat euclidean diana	
	SMOOTH euclidean average	
	RATIO euclidean diana	
10	MR sim average	1
	MR sim weighted	

Table S 12. Stable class discovery methods and associated p-value for data set **veltman**, partitions from 2 to 10 clusters, resampling rate of 80%, similarity coefficient Rogers-Tanimoto and similarity threshold $s^0 = 0.90$.

set = veltman		
resampling rate=80%, similarity coefficient=Rogers-Tanimoto, $s^0 = 0.95$		
**nb.clusters**		**p-value**
2	MR pearson average	0.0603
	MR pearson ward	
	MR pearson weighted	
	MR pearson kcentroids	
	MR euclidean diana	
	MR manhattan diana	
3	MR pearson average	0.5352
	MR pearson weighted	
4	MR euclidean average	0.0922
	MR manhattan average	
	RATIO euclidean average	
	STATUS euclidean average	
	RATIO euclidean diana	
	SMOOTH euclidean average	
	RATIO manhattan diana	
	RATIO manhattan average	
	SMOOTH euclidean diana	
	SMOOTH manhattan average	
	MR euclidean diana	
	MR manhattan diana	
	SMOOTH manhattan diana	
	STATUS euclidean diana	
	STATUS euclidean weighted	
	MR manhattan kcentroids	
5	SMOOTH manhattan average	1
	STATUS euclidean average	
	MR manhattan diana	
	SMOOTH manhattan diana	
	RATIO manhattan average	
	MR euclidean diana	
	MR euclidean average	
	MR manhattan average	
	STATUS manhattan average	
6	MR euclidean average	0.1394
	MR manhattan average	
	STATUS manhattan weighted	
7	MR euclidean average	1
8	SMOOTH euclidean diana	1
9	MR sim average	1
	MR sim weighted	
	ACPrat euclidean diana	
	SMOOTH euclidean average	
	RATIO euclidean diana	
10	MR sim average	1
	MR sim weighted	

Table S 13. Stable class discovery methods and associated *p*-value for data set **veltman**, partitions from 2 to 10 clusters, resampling rate of 80%, similarity coefficient Rogers-Tanimoto and similarity threshold $s^0 = 0.95$.

set = veltman		
resampling rate=80%, similarity coefficient=Rogers-Tanimoto, $s^0 = 0.97$		
**nb.clusters**		**p-value**
2	MR pearson average	0.0603
	MR pearson ward	
	MR pearson weighted	
	MR pearson kcentroids	
	MR euclidean diana	
	MR manhattan diana	
3	MR pearson average	0.5352
	MR pearson weighted	
4	MR euclidean average	0.0922
	MR manhattan average	
	RATIO euclidean average	
	STATUS euclidean average	
	RATIO euclidean diana	
	SMOOTH euclidean average	
	RATIO manhattan diana	
	RATIO manhattan average	
	SMOOTH euclidean diana	
	SMOOTH manhattan average	
	MR euclidean diana	
	MR manhattan diana	
	SMOOTH manhattan diana	
	STATUS euclidean diana	
	STATUS euclidean weighted	
	MR manhattan kcentroids	
5	SMOOTH manhattan average	1
	STATUS euclidean average	
	MR manhattan diana	
	SMOOTH manhattan diana	
	RATIO manhattan average	
	MR euclidean diana	
	MR euclidean average	
	MR manhattan average	
	STATUS manhattan average	
6	MR euclidean average	0.1394
	MR manhattan average	
	STATUS manhattan weighted	
7	MR euclidean average	1
8	SMOOTH euclidean diana	1
9	MR sim average	1
	MR sim weighted	
	ACPrat euclidean diana	
	SMOOTH euclidean average	
	RATIO euclidean diana	
10	MR sim average	1
	MR sim weighted	

Table S 14. Stable class discovery methods and associated p-value for data set **veltman**, partitions from 2 to 10 clusters, resampling rate of 80%, similarity coefficient Rogers-Tanimoto and similarity threshold $s^0 = 0.97$.

**set = veltman**		
resampling rate=80%, similarity coefficient=Rogers-Tanimoto, $s^0 = 0.99$		
**nb.clusters**		**p-value**
2	MR pearson average	0.0603
	MR pearson ward	
	MR pearson weighted	
	MR pearson kcentroids	
	MR euclidean diana	
	MR manhattan diana	
3	MR pearson average	0.5352
	MR pearson weighted	
4	MR euclidean average	0.0922
	MR manhattan average	
	RATIO euclidean average	
	STATUS euclidean average	
	RATIO euclidean diana	
	SMOOTH euclidean average	
	RATIO manhattan diana	
	RATIO manhattan average	
	SMOOTH euclidean diana	
	SMOOTH manhattan average	
	MR euclidean diana	
	MR manhattan diana	
	SMOOTH manhattan diana	
	STATUS euclidean diana	
	STATUS euclidean weighted	
	MR manhattan kcentroids	
5	SMOOTH manhattan average	1
	STATUS euclidean average	
	MR manhattan diana	
	SMOOTH manhattan diana	
	RATIO manhattan average	
	MR euclidean diana	
	MR euclidean average	
	MR manhattan average	
	STATUS manhattan average	
6	MR euclidean average	0.1394
	MR manhattan average	
	STATUS manhattan weighted	
7	MR euclidean average	1
8	SMOOTH euclidean diana	1
9	MR sim average	1
	MR sim weighted	
	ACPrat euclidean diana	
	SMOOTH euclidean average	
	RATIO euclidean diana	
10	MR sim average	1
	MR sim weighted	

Table S 15. Stable class discovery methods and associated p-value for data set **veltman**, partitions from 2 to 10 clusters, resampling rate of 80%, similarity coefficient Rogers-Tanimoto and similarity threshold $s^0 = 0.99$.

set = douglas		
resampling rate=80%, similarity coefficient=Jaccard, $s^0 = 0.85$		
**nb.clusters**		**p-value**
2	MR pearson average	0.6645
	MR sim average	
	MR pearson weighted	
	MR sim weighted	
	STATUS pearson average	
3	MR pearson average	0.1112
	MR agree average	
	MR conc average	
	MR sim average	
	MR pearson weighted	
	STATUS sim average	
	MR sim weighted	
4	MR sim diana	0.0799
	MR sim average	
	STATUS agree average	
	MR agree average	
	STATUS sim average	
	PCA euclidean average	
	MR conc average	
5	MR sim diana	0.0748
	MR agree average	
	MR sim average	
	SMOOTH manhattan average	
	MR sim weighted	
	STATUS agree average	
	STATUS sim diana	
	MR conc average	
	STATUS sim average	
	PCA manhattan average	
6	MR sim weighted	1
7	MR sim diana	0.0563
	MR sim weighted	
	STATUS sim average	
	MR sim average	
	STATUS agree average	
	MR agree average	
8	MR sim average	0.0985
	MR sim weighted	
	STATUS agree average	
	MR sim diana	
	MR agree average	
	STATUS sim average	

Table S 16. Stable class discovery methods and associated p-value for data set **douglas**, partitions from 2 to 8 clusters, resampling rate of 80%, similarity coefficient Jaccard and similarity threshold $s^0 = 0.85$.

**Jaccard** $s^0 = 0.85$			
**set**	**nb.clusters**		**p-value**
**douglas**			
	9	MR sim weighted	0.1134
		STATUS agree average	
		MR sim average	
		MR agree average	
		MR agree complete	
	10	MR sim average	0.1833
		MR sim weighted	
		MR agree average	
		STATUS agree average	

Table S 17. Stable class discovery methods and associated p-value for data set **douglas**, partitions from 9 to 10 clusters, resampling rate of 80%, similarity coefficient Jaccard and similarity threshold $s^0 = 0.85$.

**Jaccard** $s^0 = 0.90$			
**set**	**nb.clusters**		**p-value**
**douglas**			
	2	MR pearson average	0.6645
		MR sim average	
		MR pearson weighted	
		MR sim weighted	
		STATUS pearson average	
	3	MR pearson average	0.1112
		MR agree average	
		MR conc average	
		MR sim average	
		MR pearson weighted	
		STATUS sim average	
		MR sim weighted	
	4	MR sim diana	0.0799
		MR sim average	
		STATUS agree average	
		MR agree average	
		STATUS sim average	
		PCA euclidean average	
		MR conc average	
	5	MR sim diana	0.0748
		MR agree average	
		MR sim average	
		SMOOTH manhattan average	
		MR sim weighted	
		STATUS agree average	
		STATUS sim diana	
		MR conc average	
		STATUS sim average	
		PCA manhattan average	
	6	MR sim weighted	1
	7	MR sim diana	0.0563
		MR sim weighted	
		STATUS sim average	
		MR sim average	
		STATUS agree average	
		MR agree average	
	8	MR sim average	0.0985
		MR sim weighted	
		STATUS agree average	
		MR sim diana	
		MR agree average	
		STATUS sim average	

Table S 18. Stable class discovery methods and associated p-value for data set **douglas**, partitions from 2 to 8 clusters, resampling rate of 80%, similarity coefficient Jaccard and similarity threshold $s^0 = 0.90$.

**Jaccard** $s^0 = 0.90$			
**set**	**nb.clusters**		**p-value**
**douglas**			
	9	MR sim weighted	0.1134
		STATUS agree average	
		MR sim average	
		MR agree average	
		MR agree complete	
	10	MR sim average	0.1833
		MR sim weighted	
		MR agree average	
		STATUS agree average	

Table S 19. Stable class discovery methods and associated p-value for data set **douglas**, partitions from 9 to 10 clusters, resampling rate of 80%, similarity coefficient Jaccard and similarity threshold $s^0 = 0.90$.

Jaccard $s^0 = 0.95$			
**set**	**nb.clusters**		**p-value**
**douglas**			
	2	MR pearson average	0.6645
		MR sim average	
		MR pearson weighted	
		MR sim weighted	
		STATUS pearson average	
	3	MR pearson average	0.1112
		MR agree average	
		MR conc average	
		MR sim average	
		MR pearson weighted	
		STATUS sim average	
		MR sim weighted	
	4	MR sim diana	0.0799
		MR sim average	
		STATUS agree average	
		MR agree average	
		STATUS sim average	
		PCA euclidean average	
		MR conc average	
	5	MR sim diana	0.0748
		MR agree average	
		MR sim average	
		SMOOTH manhattan average	
		MR sim weighted	
		STATUS agree average	
		STATUS sim diana	
		MR conc average	
		STATUS sim average	
		PCA manhattan average	
	6	MR sim weighted	1
	7	MR sim diana	0.0563
		MR sim weighted	
		STATUS sim average	
		MR sim average	
		STATUS agree average	
		MR agree average	
	8	MR sim average	0.0985
		MR sim weighted	
		STATUS agree average	
		MR sim diana	
		MR agree average	
		STATUS sim average	

Table S 20. Stable class discovery methods and associated p-value for data set **douglas**, partitions from 2 to 8 clusters, resampling rate of 80%, similarity coefficient Jaccard and similarity threshold $s^0 = 0.95$.

**Jaccard** $s^0 = 0.95$			
**set**	**nb.clusters**		**p-value**
**douglas**			
	9	MR sim weighted	0.1134
		STATUS agree average	
		MR sim average	
		MR agree average	
		MR agree complete	
	10	MR sim average	0.1833
		MR sim weighted	
		MR agree average	
		STATUS agree average	

Table S 21. Stable class discovery methods and associated p-value for data set **douglas**, partitions from 9 to 10 clusters, resampling rate of 80%, similarity coefficient Jaccard and similarity threshold $s^0 = 0.95$.

**Jaccard** $s^0 = 0.97$			
**set**	**nb.clusters**		**p-value**
**douglas**			
	2	MR pearson average	0.6645
		MR sim average	
		MR pearson weighted	
		MR sim weighted	
		STATUS pearson average	
	3	MR pearson average	0.1112
		MR agree average	
		MR conc average	
		MR sim average	
		MR pearson weighted	
		STATUS sim average	
		MR sim weighted	
	4	MR sim diana	0.0799
		MR sim average	
		STATUS agree average	
		MR agree average	
		STATUS sim average	
		PCA euclidean average	
		MR conc average	
	5	MR sim diana	0.0748
		MR agree average	
		MR sim average	
		SMOOTH manhattan average	
		MR sim weighted	
		STATUS agree average	
		STATUS sim diana	
		MR conc average	
		STATUS sim average	
		PCA manhattan average	
	6	MR sim weighted	1
	7	MR sim diana	0.0563
		MR sim weighted	
		STATUS sim average	
		MR sim average	
		STATUS agree average	
		MR agree average	
	8	MR sim average	0.0985
		MR sim weighted	
		STATUS agree average	
		MR sim diana	
		MR agree average	
		STATUS sim average	

Table S 22. Stable class discovery methods and associated p-value for data set **douglas**, partitions from 2 to 8 clusters, resampling rate of 80%, similarity coefficient Jaccard and similarity threshold $s^0 = 0.97$.

**Jaccard** $s^0 = 0.97$			
**set**	**nb.clusters**		**p-value**
**douglas**			
	9	MR sim weighted	0.1134
		STATUS agree average	
		MR sim average	
		MR agree average	
		MR agree complete	
	10	MR sim average	0.1833
		MR sim weighted	
		MR agree average	
		STATUS agree average	

Table S 23. Stable class discovery methods and associated p-value for data set **douglas**, partitions from 9 to 10 clusters, resampling rate of 80%, similarity coefficient Jaccard and similarity threshold $s^0 = 0.97$.

**Jaccard** $s^0 = 0.99$			
**set**	**nb.clusters**		**p-value**
**douglas**			
	2	MR pearson average	0.6645
		MR sim average	
		MR pearson weighted	
		MR sim weighted	
		STATUS pearson average	
	3	MR pearson average	0.1112
		MR agree average	
		MR conc average	
		MR sim average	
		MR pearson weighted	
		STATUS sim average	
		MR sim weighted	
	4	MR sim diana	0.0799
		MR sim average	
		STATUS agree average	
		MR agree average	
		STATUS sim average	
		PCA euclidean average	
		MR conc average	
	5	MR sim diana	0.0748
		MR agree average	
		MR sim average	
		SMOOTH manhattan average	
		MR sim weighted	
		STATUS agree average	
		STATUS sim diana	
		MR conc average	
		STATUS sim average	
		PCA manhattan average	
	6	MR sim weighted	1
	7	MR sim diana	0.0563
		MR sim weighted	
		STATUS sim average	
		MR sim average	
		STATUS agree average	
		MR agree average	
	8	MR sim average	0.0985
		MR sim weighted	
		STATUS agree average	
		MR sim diana	
		MR agree average	
		STATUS sim average	

Table S 24. Stable class discovery methods and associated p-value for data set **douglas**, partitions from 2 to 8 clusters, resampling rate of 80%, similarity coefficient Jaccard and similarity threshold $s^0 = 0.99$.

Jaccard $s^0 = 0.99$			
**set**	**nb.clusters**		**p-value**
**douglas**			
	9	MR sim weighted	0.1134
		STATUS agree average	
		MR sim average	
		MR agree average	
		MR agree complete	
	10	MR sim average	0.1833
		MR sim weighted	
		MR agree average	
		STATUS agree average	

Table S 25. Stable class discovery methods and associated p-value for data set **douglas**, partitions from 9 to 10 clusters, resampling rate of 80%, similarity coefficient Jaccard and similarity threshold $s^0 = 0.99$.

**Simple Matching** $s^0 = 0.85$			
**set**	**nb.clusters**		**p-value**
**douglas**			
	2	MR pearson average	0.6645
		MR sim average	
		MR pearson weighted	
		MR sim weighted	
		STATUS pearson average	
	3	MR pearson average	0.1112
		MR agree average	
		MR conc average	
		MR sim average	
		MR pearson weighted	
		STATUS sim average	
		MR sim weighted	
	4	MR sim diana	0.0799
		MR sim average	
		STATUS agree average	
		MR agree average	
		STATUS sim average	
		PCA euclidean average	
		MR conc average	
	5	MR sim diana	0.0748
		MR agree average	
		MR sim average	
		SMOOTH manhattan average	
		MR sim weighted	
		STATUS agree average	
		STATUS sim diana	
		MR conc average	
		STATUS sim average	
		PCA manhattan average	
	6	MR sim weighted	1
	7	MR sim diana	0.0563
		MR sim weighted	
		STATUS sim average	
		MR sim average	
		STATUS agree average	
		MR agree average	
	8	MR sim average	0.0985
		MR sim weighted	
		STATUS agree average	
		MR sim diana	
		MR agree average	
		STATUS sim average	

Table S 26. Stable class discovery methods and associated p-value for data set **douglas**, partitions from 2 to 8 clusters, resampling rate of 80%, similarity coefficient Simple Matching and similarity threshold $s^0 = 0.85$.

Simple Matching $s^0 = 0.85$			
**set**	**nb.clusters**		**p-value**
**douglas**			
	9	MR sim weighted<br>STATUS agree average<br>MR sim average<br>MR agree average<br>MR agree complete	0.1134
	10	MR sim average<br>MR sim weighted<br>MR agree average<br>STATUS agree average	0.1833

Table S 27. Stable class discovery methods and associated p-value for data set **douglas**, partitions from 9 to 10 clusters, resampling rate of 80%, similarity coefficient Simple Matching and similarity threshold $s^0 = 0.85$.

**Simple Matching** $s^0 = 0.90$			
**set**	**nb.clusters**		**p-value**
**douglas**			
	2	MR pearson average	0.6645
		MR sim average	
		MR pearson weighted	
		MR sim weighted	
		STATUS pearson average	
	3	MR pearson average	0.1112
		MR agree average	
		MR conc average	
		MR sim average	
		MR pearson weighted	
		STATUS sim average	
		MR sim weighted	
	4	MR sim diana	0.0799
		MR sim average	
		STATUS agree average	
		MR agree average	
		STATUS sim average	
		PCA euclidean average	
		MR conc average	
	5	MR sim diana	0.0748
		MR agree average	
		MR sim average	
		SMOOTH manhattan average	
		MR sim weighted	
		STATUS agree average	
		STATUS sim diana	
		MR conc average	
		STATUS sim average	
		PCA manhattan average	
	6	MR sim weighted	1
	7	MR sim diana	0.0563
		MR sim weighted	
		STATUS sim average	
		MR sim average	
		STATUS agree average	
		MR agree average	
	8	MR sim average	0.0985
		MR sim weighted	
		STATUS agree average	
		MR sim diana	
		MR agree average	
		STATUS sim average	

Table S 28. Stable class discovery methods and associated p-value for data set **douglas**, partitions from 2 to 8 clusters, resampling rate of 80%, similarity coefficient Simple Matching and similarity threshold $s^0 = 0.90$.

**Simple Matching** $s^0 = 0.90$			
**set**	**nb.clusters**		**p-value**
**douglas**			
	9	MR sim weighted<br>STATUS agree average<br>MR sim average<br>MR agree average<br>MR agree complete	0.1134
	10	MR sim average<br>MR sim weighted<br>MR agree average<br>STATUS agree average	0.1833

Table S 29. Stable class discovery methods and associated p-value for data set **douglas**, partitions from 9 to 10 clusters, resampling rate of 80%, similarity coefficient Simple Matching and similarity threshold $s^0 = 0.90$.

**Simple Matching** $s^0 = 0.95$			
**set**	**nb.clusters**		**p-value**
**douglas**			
	2	MR pearson average	0.6645
		MR sim average	
		MR pearson weighted	
		MR sim weighted	
		STATUS pearson average	
	3	MR pearson average	0.1112
		MR agree average	
		MR conc average	
		MR sim average	
		MR pearson weighted	
		STATUS sim average	
		MR sim weighted	
	4	MR sim diana	0.0799
		MR sim average	
		STATUS agree average	
		MR agree average	
		STATUS sim average	
		PCA euclidean average	
		MR conc average	
	5	MR sim diana	0.0748
		MR agree average	
		MR sim average	
		SMOOTH manhattan average	
		MR sim weighted	
		STATUS agree average	
		STATUS sim diana	
		MR conc average	
		STATUS sim average	
		PCA manhattan average	
	6	MR sim weighted	1
	7	MR sim diana	0.0563
		MR sim weighted	
		STATUS sim average	
		MR sim average	
		STATUS agree average	
		MR agree average	
	8	MR sim average	0.0985
		MR sim weighted	
		STATUS agree average	
		MR sim diana	
		MR agree average	
		STATUS sim average	

Table S 30. Stable class discovery methods and associated p-value for data set **douglas**, partitions from 2 to 8 clusters, resampling rate of 80%, similarity coefficient Simple Matching and similarity threshold $s^0 = 0.95$.

**Simple Matching** $s^0 = 0.95$			
**set**	**nb.clusters**		**p-value**
**douglas**			
	9	MR sim weighted<br>STATUS agree average<br>MR sim average<br>MR agree average<br>MR agree complete	0.1134
	10	MR sim average<br>MR sim weighted<br>MR agree average<br>STATUS agree average	0.1833

Table S 31. Stable class discovery methods and associated p-value for data set **douglas**, partitions from 9 to 10 clusters, resampling rate of 80%, similarity coefficient Simple Matching and similarity threshold $s^0 = 0.95$.

Simple Matching $s^0 = 0.97$			
**set**	**nb.clusters**		**p-value**
**douglas**			
	2	MR pearson average	0.6645
		MR sim average	
		MR pearson weighted	
		MR sim weighted	
		STATUS pearson average	
	3	MR pearson average	0.1112
		MR agree average	
		MR conc average	
		MR sim average	
		MR pearson weighted	
		STATUS sim average	
		MR sim weighted	
	4	MR sim diana	0.0799
		MR sim average	
		STATUS agree average	
		MR agree average	
		STATUS sim average	
		PCA euclidean average	
		MR conc average	
	5	MR sim diana	0.0748
		MR agree average	
		MR sim average	
		SMOOTH manhattan average	
		MR sim weighted	
		STATUS agree average	
		STATUS sim diana	
		MR conc average	
		STATUS sim average	
		PCA manhattan average	
	6	MR sim weighted	1
	7	MR sim diana	0.0563
		MR sim weighted	
		STATUS sim average	
		MR sim average	
		STATUS agree average	
		MR agree average	
	8	MR sim average	0.0985
		MR sim weighted	
		STATUS agree average	
		MR sim diana	
		MR agree average	
		STATUS sim average	

Table S 32. Stable class discovery methods and associated p-value for data set **douglas**, partitions from 2 to 8 clusters, resampling rate of 80%, similarity coefficient Simple Matching and similarity threshold $s^0 = 0.97$.

**Simple Matching** $s^0 = 0.97$			
**set**	**nb.clusters**		**p-value**
**douglas**			
	9	MR sim weighted<br>STATUS agree average<br>MR sim average<br>MR agree average<br>MR agree complete	0.1134
	10	MR sim average<br>MR sim weighted<br>MR agree average<br>STATUS agree average	0.1833

Table S 33. Stable class discovery methods and associated p-value for data set **douglas**, partitions from 9 to 10 clusters, resampling rate of 80%, similarity coefficient Simple Matching and similarity threshold $s^0 = 0.97$.

Simple Matching $s^0 = 0.99$			
**set**	**nb.clusters**		**p-value**
**douglas**			
	2	MR pearson average	0.6645
		MR sim average	
		MR pearson weighted	
		MR sim weighted	
		STATUS pearson average	
	3	MR pearson average	0.1112
		MR agree average	
		MR conc average	
		MR sim average	
		MR pearson weighted	
		STATUS sim average	
		MR sim weighted	
	4	MR sim diana	0.0799
		MR sim average	
		STATUS agree average	
		MR agree average	
		STATUS sim average	
		PCA euclidean average	
		MR conc average	
	5	MR sim diana	0.0748
		MR agree average	
		MR sim average	
		SMOOTH manhattan average	
		MR sim weighted	
		STATUS agree average	
		STATUS sim diana	
		MR conc average	
		STATUS sim average	
		PCA manhattan average	
	6	MR sim weighted	1
	7	MR sim diana	0.0563
		MR sim weighted	
		STATUS sim average	
		MR sim average	
		STATUS agree average	
		MR agree average	
	8	MR sim average	0.0985
		MR sim weighted	
		STATUS agree average	
		MR sim diana	
		MR agree average	
		STATUS sim average	

Table S 34. Stable class discovery methods and associated p-value for data set **douglas**, partitions from 2 to 8 clusters, resampling rate of 80%, similarity coefficient Simple Matching and similarity threshold $s^0 = 0.99$.

**Simple Matching** $s^0 = 0.99$			
**set**	**nb.clusters**		**p-value**
**douglas**			
	9	MR sim weighted	0.1134
		STATUS agree average	
		MR sim average	
		MR agree average	
		MR agree complete	
	10	MR sim average	0.1833
		MR sim weighted	
		MR agree average	
		STATUS agree average	

Table S 35. Stable class discovery methods and associated p-value for data set **douglas**, partitions from 9 to 10 clusters, resampling rate of 80%, similarity coefficient Simple Matching and similarity threshold $s^0 = 0.99$.

Rogers-Tanimoto $s^0 = 0.85$			
**set**	**nb.clusters**		**p-value**
**douglas**			
	2	MR pearson average	0.6645
		MR sim average	
		MR pearson weighted	
		MR sim weighted	
		STATUS pearson average	
	3	MR pearson average	0.1112
		MR agree average	
		MR conc average	
		MR sim average	
		MR pearson weighted	
		STATUS sim average	
		MR sim weighted	
	4	MR sim diana	0.0799
		MR sim average	
		STATUS agree average	
		MR agree average	
		STATUS sim average	
		PCA euclidean average	
		MR conc average	
	5	MR sim diana	0.0748
		MR agree average	
		MR sim average	
		SMOOTH manhattan average	
		MR sim weighted	
		STATUS agree average	
		STATUS sim diana	
		MR conc average	
		STATUS sim average	
		PCA manhattan average	
	6	MR sim weighted	1
	7	MR sim diana	0.0563
		MR sim weighted	
		STATUS sim average	
		MR sim average	
		STATUS agree average	
		MR agree average	
	8	MR sim average	0.0985
		MR sim weighted	
		STATUS agree average	
		MR sim diana	
		MR agree average	
		STATUS sim average	

Table S 36. Stable class discovery methods and associated p-value for data set **douglas**, partitions from 2 to 8 clusters, resampling rate of 80%, similarity coefficient Rogers-Tanimoto and similarity threshold $s^0 = 0.85$.

Rogers-Tanimoto $s^0 = 0.85$			
**set**	**nb.clusters**		**p-value**
**douglas**			
	9	MR sim weighted<br>STATUS agree average<br>MR sim average<br>MR agree average<br>MR agree complete	0.1134
	10	MR sim average<br>MR sim weighted<br>MR agree average<br>STATUS agree average	0.1833

Table S 37. Stable class discovery methods and associated p-value for data set **douglas**, partitions from 9 to 10 clusters, resampling rate of 80%, similarity coefficient Rogers-Tanimoto and similarity threshold $s^0 = 0.85$.

Rogers-Tanimoto $s^0 = 0.90$			
**set**	**nb.clusters**		**p-value**
**douglas**			
	2	MR pearson average	0.6645
		MR sim average	
		MR pearson weighted	
		MR sim weighted	
		STATUS pearson average	
	3	MR pearson average	0.1112
		MR agree average	
		MR conc average	
		MR sim average	
		MR pearson weighted	
		STATUS sim average	
		MR sim weighted	
	4	MR sim diana	0.0799
		MR sim average	
		STATUS agree average	
		MR agree average	
		STATUS sim average	
		PCA euclidean average	
		MR conc average	
	5	MR sim diana	0.0748
		MR agree average	
		MR sim average	
		SMOOTH manhattan average	
		MR sim weighted	
		STATUS agree average	
		STATUS sim diana	
		MR conc average	
		STATUS sim average	
		PCA manhattan average	
	6	MR sim weighted	1
	7	MR sim diana	0.0563
		MR sim weighted	
		STATUS sim average	
		MR sim average	
		STATUS agree average	
		MR agree average	
	8	MR sim average	0.0985
		MR sim weighted	
		STATUS agree average	
		MR sim diana	
		MR agree average	
		STATUS sim average	

Table S 38. Stable class discovery methods and associated p-value for data set **douglas**, partitions from 2 to 8 clusters, resampling rate of 80%, similarity coefficient Rogers-Tanimoto and similarity threshold $s^0 = 0.90$.

Rogers-Tanimoto $s^0 = 0.90$			
**set**	**nb.clusters**		**p-value**
**douglas**			
	9	MR sim weighted<br>STATUS agree average<br>MR sim average<br>MR agree average<br>MR agree complete	0.1134
	10	MR sim average<br>MR sim weighted<br>MR agree average<br>STATUS agree average	0.1833

Table S 39. Stable class discovery methods and associated p-value for data set **douglas**, partitions from 9 to 10 clusters, resampling rate of 80%, similarity coefficient Rogers-Tanimoto and similarity threshold $s^0 = 0.90$.

**Rogers-Tanimoto** $s^0 = 0.95$			
**set**	**nb.clusters**		**p-value**
**douglas**			
	2	MR pearson average	0.6645
		MR sim average	
		MR pearson weighted	
		MR sim weighted	
		STATUS pearson average	
	3	MR pearson average	0.1112
		MR agree average	
		MR conc average	
		MR sim average	
		MR pearson weighted	
		STATUS sim average	
		MR sim weighted	
	4	MR sim diana	0.0799
		MR sim average	
		STATUS agree average	
		MR agree average	
		STATUS sim average	
		PCA euclidean average	
		MR conc average	
	5	MR sim diana	0.0748
		MR agree average	
		MR sim average	
		SMOOTH manhattan average	
		MR sim weighted	
		STATUS agree average	
		STATUS sim diana	
		MR conc average	
		STATUS sim average	
		PCA manhattan average	
	6	MR sim weighted	1
	7	MR sim diana	0.0563
		MR sim weighted	
		STATUS sim average	
		MR sim average	
		STATUS agree average	
		MR agree average	
	8	MR sim average	0.0985
		MR sim weighted	
		STATUS agree average	
		MR sim diana	
		MR agree average	
		STATUS sim average	

Table S 40. Stable class discovery methods and associated p-value for data set **douglas**, partitions from 2 to 8 clusters, resampling rate of 80%, similarity coefficient Rogers-Tanimoto and similarity threshold $s^0 = 0.95$.

**Rogers-Tanimoto** $s^0 = 0.95$			
**set**	**nb.clusters**		**p-value**
**douglas**			
	9	MR sim weighted<br>STATUS agree average<br>MR sim average<br>MR agree average<br>MR agree complete	0.1134
	10	MR sim average<br>MR sim weighted<br>MR agree average<br>STATUS agree average	0.1833

Table S 41. Stable class discovery methods and associated p-value for data set **douglas**, partitions from 9 to 10 clusters, resampling rate of 80%, similarity coefficient Rogers-Tanimoto and similarity threshold $s^0 = 0.95$.

Rogers-Tanimoto $s^0 = 0.97$			
**set**	**nb.clusters**		**p-value**
**douglas**			
	2	MR pearson average	0.6645
		MR sim average	
		MR pearson weighted	
		MR sim weighted	
		STATUS pearson average	
	3	MR pearson average	0.1112
		MR agree average	
		MR conc average	
		MR sim average	
		MR pearson weighted	
		STATUS sim average	
		MR sim weighted	
	4	MR sim diana	0.0799
		MR sim average	
		STATUS agree average	
		MR agree average	
		STATUS sim average	
		PCA euclidean average	
		MR conc average	
	5	MR sim diana	0.0748
		MR agree average	
		MR sim average	
		SMOOTH manhattan average	
		MR sim weighted	
		STATUS agree average	
		STATUS sim diana	
		MR conc average	
		STATUS sim average	
		PCA manhattan average	
	6	MR sim weighted	1
	7	MR sim diana	0.0563
		MR sim weighted	
		STATUS sim average	
		MR sim average	
		STATUS agree average	
		MR agree average	
	8	MR sim average	0.0985
		MR sim weighted	
		STATUS agree average	
		MR sim diana	
		MR agree average	
		STATUS sim average	

Table S 42. Stable class discovery methods and associated p-value for data set **douglas**, partitions from 2 to 8 clusters, resampling rate of 80%, similarity coefficient Rogers-Tanimoto and similarity threshold $s^0 = 0.97$.

**Rogers-Tanimoto** $s^0 = 0.97$			
**set**	**nb.clusters**		**p-value**
**douglas**			
	9	MR sim weighted<br>STATUS agree average<br>MR sim average<br>MR agree average<br>MR agree complete	0.1134
	10	MR sim average<br>MR sim weighted<br>MR agree average<br>STATUS agree average	0.1833

Table S 43. Stable class discovery methods and associated p-value for data set **douglas**, partitions from 9 to 10 clusters, resampling rate of 80%, similarity coefficient Rogers-Tanimoto and similarity threshold $s^0 = 0.97$.

**Rogers-Tanimoto** $s^0 = 0.99$			
**set**	**nb.clusters**		**p-value**
**douglas**			
	2	MR pearson average	0.6645
		MR sim average	
		MR pearson weighted	
		MR sim weighted	
		STATUS pearson average	
	3	MR pearson average	0.1112
		MR agree average	
		MR conc average	
		MR sim average	
		MR pearson weighted	
		STATUS sim average	
		MR sim weighted	
	4	MR sim diana	0.0799
		MR sim average	
		STATUS agree average	
		MR agree average	
		STATUS sim average	
		PCA euclidean average	
		MR conc average	
	5	MR sim diana	0.0748
		MR agree average	
		MR sim average	
		SMOOTH manhattan average	
		MR sim weighted	
		STATUS agree average	
		STATUS sim diana	
		MR conc average	
		STATUS sim average	
		PCA manhattan average	
	6	MR sim weighted	1
	7	MR sim diana	0.0563
		MR sim weighted	
		STATUS sim average	
		MR sim average	
		STATUS agree average	
		MR agree average	
	8	MR sim average	0.0985
		MR sim weighted	
		STATUS agree average	
		MR sim diana	
		MR agree average	
		STATUS sim average	

Table S 44. Stable class discovery methods and associated p-value for data set **douglas**, partitions from 2 to 8 clusters, resampling rate of 80%, similarity coefficient Rogers-Tanimoto and similarity threshold $s^0 = 0.99$.

Rogers-Tanimoto $s^0 = 0.99$			
**set**	**nb.clusters**		**p-value**
**douglas**			
	9	MR sim weighted<br>STATUS agree average<br>MR sim average<br>MR agree average<br>MR agree complete	0.1134
	10	MR sim average<br>MR sim weighted<br>MR agree average<br>STATUS agree average	0.1833

Table S 45. Stable class discovery methods and associated p-value for data set **douglas**, partitions from 9 to 10 clusters, resampling rate of 80%, similarity coefficient Rogers-Tanimoto and similarity threshold $s^0 = 0.99$.

Jaccard $s^0 = 0.85$			
**set**	**nb.clusters**		**p-value**
**gysin**			
	2	MR sim average	0.3146
		STATUS sim diana	
		MR agree average	
		MR sim diana	
		MR sim weighted	
		STATUS sim average	
		MR pearson average	
		RATIO euclidean ward	
		PCA euclidean weighted	
		RATIO euclidean tsvq	
		PCA euclidean average	
	3	MR agree average	0.1446
		MR sim average	
		STATUS sim average	
		STATUS sim diana	
		STATUS agree average	
		MR sim weighted	
		STATUS sim weighted	
	4	MR agree average	0.2418
		STATUS agree average	
		MR sim average	
		STATUS sim average	
		MR agree complete	
		MR sim weighted	
	5	MR agree average	0.0772
		STATUS agree average	
		MR agree complete	
		MR sim weighted	
		MR sim average	
	6	MR agree average	0.2372
		STATUS agree average	
		MR agree complete	
		MR sim average	
		MR sim weighted	
		PCA euclidean average	
	7	MR agree average	1
		MR agree complete	
		MR sim average	
		MR sim weighted	
		STATUS agree average	
	8	MR agree average	0.7509
		MR conc average	
		STATUS agree average	
		MR agree complete	
		MR sim weighted	
		MR sim average	

Table S 46. Stable class discovery methods and associated p-value for data set **gysin**, partitions from 2 to 8 clusters, similarity coefficient Jaccard and similarity threshold $s^0 = 0.85$.

**Jaccard** $s^0 = 0.85$			
**set**	**nb.clusters**		**p-value**
**gysin**			
	9	MR agree average<br>MR agree complete<br>STATUS agree average	1
	10	STATUS agree average<br>MR agree average	0.0970

Table S 47. Stable class discovery methods and associated p-value for data set **gysin**, partitions from 9 to 10 clusters, similarity coefficient Jaccard and similarity threshold $s^0 = 0.85$.

**Jaccard** $s^0 = 0.90$			
**set**	**nb.clusters**		**p-value**
**gysin**			
	2	MR sim average	0.3146
		STATUS sim diana	
		MR agree average	
		MR sim diana	
		MR sim weighted	
		STATUS sim average	
		MR pearson average	
		RATIO euclidean ward	
		PCA euclidean weighted	
		RATIO euclidean tsvq	
		PCA euclidean average	
	3	MR agree average	0.1446
		MR sim average	
		STATUS sim average	
		STATUS sim diana	
		STATUS agree average	
		MR sim weighted	
		STATUS sim weighted	
	4	MR agree average	0.2418
		STATUS agree average	
		MR sim average	
		STATUS sim average	
		MR agree complete	
		MR sim weighted	
	5	MR agree average	0.0772
		STATUS agree average	
		MR agree complete	
		MR sim weighted	
		MR sim average	
	6	MR agree average	0.2372
		STATUS agree average	
		MR agree complete	
		MR sim average	
		MR sim weighted	
		PCA euclidean average	
	7	MR agree average	1
		MR agree complete	
		MR sim average	
		MR sim weighted	
		STATUS agree average	
	8	MR agree average	0.7509
		MR conc average	
		STATUS agree average	
		MR agree complete	
		MR sim weighted	
		MR sim average	

Table S 48. Stable class discovery methods and associated p-value for data set **gysin**, partitions from 2 to 8 clusters, similarity coefficient Jaccard and similarity threshold $s^0 = 0.90$.

**Jaccard** $s^0 = 0.90$			
**set**	**nb.clusters**		**p-value**
**gysin**			
	9	MR agree average	1
		MR agree complete	
		STATUS agree average	
	10	STATUS agree average	0.0970
		MR agree average	

Table S 49. Stable class discovery methods and associated p-value for data set **gysin**, partitions from 9 to 10 clusters, similarity coefficient Jaccard and similarity threshold $s^0 = 0.90$.

Jaccard $s^0 = 0.95$			
**set**	**nb.clusters**		**p-value**
**gysin**			
	2	MR sim average	0.3146
		STATUS sim diana	
		MR agree average	
		MR sim diana	
		MR sim weighted	
		STATUS sim average	
		MR pearson average	
		RATIO euclidean ward	
		PCA euclidean weighted	
		RATIO euclidean tsvq	
		PCA euclidean average	
	3	MR agree average	0.1446
		MR sim average	
		STATUS sim average	
		STATUS sim diana	
		STATUS agree average	
		MR sim weighted	
		STATUS sim weighted	
	4	MR agree average	0.2418
		STATUS agree average	
		MR sim average	
		STATUS sim average	
		MR agree complete	
		MR sim weighted	
	5	MR agree average	0.0772
		STATUS agree average	
		MR agree complete	
		MR sim weighted	
		MR sim average	
	6	MR agree average	0.2372
		STATUS agree average	
		MR agree complete	
		MR sim average	
		MR sim weighted	
		PCA euclidean average	
	7	MR agree average	1
		MR agree complete	
		MR sim average	
		MR sim weighted	
		STATUS agree average	
	8	MR agree average	0.7509
		MR conc average	
		STATUS agree average	
		MR agree complete	
		MR sim weighted	
		MR sim average	

Table S 50. Stable class discovery methods and associated p-value for data set **gysin**, partitions from 2 to 8 clusters, similarity coefficient Jaccard and similarity threshold $s^0 = 0.95$.

**Jaccard** $s^0 = 0.95$			
**set**	**nb.clusters**		**p-value**
**gysin**			
	9	MR agree average	1
		MR agree complete	
		STATUS agree average	
	10	STATUS agree average	0.0970
		MR agree average	

Table S 51. Stable class discovery methods and associated p-value for data set **gysin**, partitions from 9 to 10 clusters, similarity coefficient Jaccard and similarity threshold $s^0 = 0.95$.

Jaccard $s^0 = 0.97$			
set	nb.clusters		p-value
gysin			
	2	MR sim average	0.3146
		STATUS sim diana	
		MR agree average	
		MR sim diana	
		MR sim weighted	
		STATUS sim average	
		MR pearson average	
		RATIO euclidean ward	
		PCA euclidean weighted	
		RATIO euclidean tsvq	
		PCA euclidean average	
	3	MR agree average	0.1446
		MR sim average	
		STATUS sim average	
		STATUS sim diana	
		STATUS agree average	
		MR sim weighted	
		STATUS sim weighted	
	4	MR agree average	0.2418
		STATUS agree average	
		MR sim average	
		STATUS sim average	
		MR agree complete	
		MR sim weighted	
	5	MR agree average	0.0772
		STATUS agree average	
		MR agree complete	
		MR sim weighted	
		MR sim average	
	6	MR agree average	0.2372
		STATUS agree average	
		MR agree complete	
		MR sim average	
		MR sim weighted	
		PCA euclidean average	
	7	MR agree average	1
		MR agree complete	
		MR sim average	
		MR sim weighted	
		STATUS agree average	
	8	MR agree average	0.7509
		MR conc average	
		STATUS agree average	
		MR agree complete	
		MR sim weighted	
		MR sim average	

Table S 52. Stable class discovery methods and associated p-value for data set **gysin**, partitions from 2 to 8 clusters, similarity coefficient Jaccard and similarity threshold $s^0 = 0.97$.

**Jaccard** $s^0 = 0.97$			
**set**	**nb.clusters**		**p-value**
**gysin**			
	9	MR agree average	1
		MR agree complete	
		STATUS agree average	
	10	STATUS agree average	0.0970
		MR agree average	

Table S 53. Stable class discovery methods and associated p-value for data set **gysin**, partitions from 9 to 10 clusters, similarity coefficient Jaccard and similarity threshold $s^0 = 0.97$.

Jaccard $s^0 = 0.99$			
**set**	**nb.clusters**		**p-value**
**gysin**			
	2	MR sim average	0.3146
		STATUS sim diana	
		MR agree average	
		MR sim diana	
		MR sim weighted	
		STATUS sim average	
		MR pearson average	
		RATIO euclidean ward	
		PCA euclidean weighted	
		RATIO euclidean tsvq	
		PCA euclidean average	
	3	MR agree average	0.1446
		MR sim average	
		STATUS sim average	
		STATUS sim diana	
		STATUS agree average	
		MR sim weighted	
		STATUS sim weighted	
	4	MR agree average	0.2418
		STATUS agree average	
		MR sim average	
		STATUS sim average	
		MR agree complete	
		MR sim weighted	
	5	MR agree average	0.0772
		STATUS agree average	
		MR agree complete	
		MR sim weighted	
		MR sim average	
	6	MR agree average	0.2372
		STATUS agree average	
		MR agree complete	
		MR sim average	
		MR sim weighted	
		PCA euclidean average	
	7	MR agree average	1
		MR agree complete	
		MR sim average	
		MR sim weighted	
		STATUS agree average	
	8	MR agree average	0.7509
		MR conc average	
		STATUS agree average	
		MR agree complete	
		MR sim weighted	
		MR sim average	

Table S 54. Stable class discovery methods and associated p-value for data set **gysin**, partitions from 2 to 8 clusters, similarity coefficient Jaccard and similarity threshold $s^0 = 0.99$.

**Jaccard** $s^0 = 0.99$			
**set**	**nb.clusters**		**p-value**
**gysin**			
	9	MR agree average	1
		MR agree complete	
		STATUS agree average	
	10	STATUS agree average	0.0970
		MR agree average	

Table S 55. Stable class discovery methods and associated p-value for data set **gysin**, partitions from 9 to 10 clusters, similarity coefficient Jaccard and similarity threshold $s^0 = 0.99$.

Simple Matching $s^0 = 0.85$			
set	nb.clusters		p-value
gysin			
	2	MR sim average<br>STATUS sim diana<br>MR agree average<br>MR sim diana<br>MR sim weighted<br>STATUS sim average<br>MR pearson average<br>RATIO euclidean ward<br>PCA euclidean weighted<br>RATIO euclidean tsvq<br>PCA euclidean average	0.3146
	3	MR agree average<br>MR sim average<br>STATUS sim average<br>STATUS sim diana<br>STATUS agree average<br>MR sim weighted<br>STATUS sim weighted	0.1446
	4	MR agree average<br>STATUS agree average<br>MR sim average<br>STATUS sim average<br>MR agree complete<br>MR sim weighted	0.2418
	5	MR agree average<br>STATUS agree average<br>MR agree complete<br>MR sim weighted<br>MR sim average	0.0772
	6	MR agree average<br>STATUS agree average<br>MR agree complete<br>MR sim average<br>MR sim weighted<br>PCA euclidean average	0.2372
	7	MR agree average<br>MR agree complete<br>MR sim average<br>MR sim weighted<br>STATUS agree average	1
	8	MR agree average<br>MR conc average<br>STATUS agree average<br>MR agree complete<br>MR sim weighted<br>MR sim average	0.7509

Table S 56. Stable class discovery methods and associated p-value for data set **gysin**, partitions from 2 to 8 clusters, similarity coefficient Simple Matching and similarity threshold $s^0 = 0.85$.

**Simple Matching** $s^0 = 0.85$			
**set**	**nb.clusters**		**p-value**
**gysin**			
	9	MR agree average<br>MR agree complete<br>STATUS agree average	1
	10	STATUS agree average<br>MR agree average	0.0970

Table S 57. Stable class discovery methods and associated p-value for data set **gysin**, partitions from 9 to 10 clusters, similarity coefficient Simple Matching and similarity threshold $s^0 = 0.85$.

**Simple Matching** $s^0 = 0.90$			
**set**	**nb.clusters**		**p-value**
**gysin**			
	2	MR sim average<br>STATUS sim diana<br>MR agree average<br>MR sim diana<br>MR sim weighted<br>STATUS sim average<br>MR pearson average<br>RATIO euclidean ward<br>PCA euclidean weighted<br>RATIO euclidean tsvq<br>PCA euclidean average	0.3146
	3	MR agree average<br>MR sim average<br>STATUS sim average<br>STATUS sim diana<br>STATUS agree average<br>MR sim weighted<br>STATUS sim weighted	0.1446
	4	MR agree average<br>STATUS agree average<br>MR sim average<br>STATUS sim average<br>MR agree complete<br>MR sim weighted	0.2418
	5	MR agree average<br>STATUS agree average<br>MR agree complete<br>MR sim weighted<br>MR sim average	0.0772
	6	MR agree average<br>STATUS agree average<br>MR agree complete<br>MR sim average<br>MR sim weighted<br>PCA euclidean average	0.2372
	7	MR agree average<br>MR agree complete<br>MR sim average<br>MR sim weighted<br>STATUS agree average	1
	8	MR agree average<br>MR conc average<br>STATUS agree average<br>MR agree complete<br>MR sim weighted<br>MR sim average	0.7509

Table S 58. Stable class discovery methods and associated p-value for data set **gysin**, partitions from 2 to 8 clusters, similarity coefficient Simple Matching and similarity threshold $s^0 = 0.90$.

**Simple Matching** $s^0 = 0.90$			
**set**	**nb.clusters**		**p-value**
**gysin**			
	9	MR agree average<br>MR agree complete<br>STATUS agree average	1
	10	STATUS agree average<br>MR agree average	0.0970

Table S 59. Stable class discovery methods and associated p-value for data set **gysin**, partitions from 9 to 10 clusters, similarity coefficient Simple Matching and similarity threshold $s^0 = 0.90$.

**Simple Matching** $s^0 = 0.95$			
**set**	**nb.clusters**		**p-value**
**gysin**			
	2	MR sim average<br>STATUS sim diana<br>MR agree average<br>MR sim diana<br>MR sim weighted<br>STATUS sim average<br>MR pearson average<br>RATIO euclidean ward<br>PCA euclidean weighted<br>RATIO euclidean tsvq<br>PCA euclidean average	0.3146
	3	MR agree average<br>MR sim average<br>STATUS sim average<br>STATUS sim diana<br>STATUS agree average<br>MR sim weighted<br>STATUS sim weighted	0.1446
	4	MR agree average<br>STATUS agree average<br>MR sim average<br>STATUS sim average<br>MR agree complete<br>MR sim weighted	0.2418
	5	MR agree average<br>STATUS agree average<br>MR agree complete<br>MR sim weighted<br>MR sim average	0.0772
	6	MR agree average<br>STATUS agree average<br>MR agree complete<br>MR sim average<br>MR sim weighted<br>PCA euclidean average	0.2372
	7	MR agree average<br>MR agree complete<br>MR sim average<br>MR sim weighted<br>STATUS agree average	1
	8	MR agree average<br>MR conc average<br>STATUS agree average<br>MR agree complete<br>MR sim weighted<br>MR sim average	0.7509

Table S 60. Stable class discovery methods and associated *p*-value for data set **gysin**, partitions from 2 to 8 clusters, similarity coefficient Simple Matching and similarity threshold $s^0 = 0.95$.

Simple Matching $s^0 = 0.95$			
**set**	**nb.clusters**		**p-value**
**gysin**			
	9	MR agree average<br>MR agree complete<br>STATUS agree average	1
	10	STATUS agree average<br>MR agree average	0.0970

Table S 61. Stable class discovery methods and associated p-value for data set **gysin**, partitions from 9 to 10 clusters, similarity coefficient Simple Matching and similarity threshold $s^0 = 0.95$.

Simple Matching $s^0 = 0.97$			
set	nb.clusters		p-value
gysin			
	2	MR sim average<br>STATUS sim diana<br>MR agree average<br>MR sim diana<br>MR sim weighted<br>STATUS sim average<br>MR pearson average<br>RATIO euclidean ward<br>PCA euclidean weighted<br>RATIO euclidean tsvq<br>PCA euclidean average	0.3146
	3	MR agree average<br>MR sim average<br>STATUS sim average<br>STATUS sim diana<br>STATUS agree average<br>MR sim weighted<br>STATUS sim weighted	0.1446
	4	MR agree average<br>STATUS agree average<br>MR sim average<br>STATUS sim average<br>MR agree complete<br>MR sim weighted	0.2418
	5	MR agree average<br>STATUS agree average<br>MR agree complete<br>MR sim weighted<br>MR sim average	0.0772
	6	MR agree average<br>STATUS agree average<br>MR agree complete<br>MR sim average<br>MR sim weighted<br>PCA euclidean average	0.2372
	7	MR agree average<br>MR agree complete<br>MR sim average<br>MR sim weighted<br>STATUS agree average	1
	8	MR agree average<br>MR conc average<br>STATUS agree average<br>MR agree complete<br>MR sim weighted<br>MR sim average	0.7509

Table S 62. Stable class discovery methods and associated p-value for data set **gysin**, partitions from 2 to 8 clusters, similarity coefficient Simple Matching and similarity threshold $s^0 = 0.97$.

**Simple Matching** $s^0 = 0.97$			
**set**	**nb.clusters**		**p-value**
**gysin**			
	9	MR agree average<br>MR agree complete<br>STATUS agree average	1
	10	STATUS agree average<br>MR agree average	0.0970

Table S 63. Stable class discovery methods and associated p-value for data set **gysin**, partitions from 9 to 10 clusters, similarity coefficient Simple Matching and similarity threshold $s^0 = 0.97$.

Simple Matching $s^0 = 0.99$			
**set**	**nb.clusters**		**p-value**
**gysin**			
	2	MR sim average<br>STATUS sim diana<br>MR agree average<br>MR sim diana<br>MR sim weighted<br>STATUS sim average<br>MR pearson average<br>RATIO euclidean ward<br>PCA euclidean weighted<br>RATIO euclidean tsvq<br>PCA euclidean average	0.3146
	3	MR agree average<br>MR sim average<br>STATUS sim average<br>STATUS sim diana<br>STATUS agree average<br>MR sim weighted<br>STATUS sim weighted	0.1446
	4	MR agree average<br>STATUS agree average<br>MR sim average<br>STATUS sim average<br>MR agree complete<br>MR sim weighted	0.2418
	5	MR agree average<br>STATUS agree average<br>MR agree complete<br>MR sim weighted<br>MR sim average	0.0772
	6	MR agree average<br>STATUS agree average<br>MR agree complete<br>MR sim average<br>MR sim weighted<br>PCA euclidean average	0.2372
	7	MR agree average<br>MR agree complete<br>MR sim average<br>MR sim weighted<br>STATUS agree average	1
	8	MR agree average<br>MR conc average<br>STATUS agree average<br>MR agree complete<br>MR sim weighted<br>MR sim average	0.7509

Table S 64. Stable class discovery methods and associated p-value for data set **gysin**, partitions from 2 to 8 clusters, similarity coefficient Simple Matching and similarity threshold $s^0 = 0.99$.

**Simple Matching** $s^0 = 0.99$			
**set**	**nb.clusters**		**p-value**
**gysin**			
	9	MR agree average	1
		MR agree complete	
		STATUS agree average	
	10	STATUS agree average	0.0970
		MR agree average	

Table S 65. Stable class discovery methods and associated p-value for data set **gysin**, partitions from 9 to 10 clusters, similarity coefficient Simple Matching and similarity threshold $s^0 = 0.99$.

**Rogers-Tanimoto** $s^0 = 0.85$			
**set**	**nb.clusters**		**p-value**
**gysin**			
	2	MR sim average	0.3146
		STATUS sim diana	
		MR agree average	
		MR sim diana	
		MR sim weighted	
		STATUS sim average	
		MR pearson average	
		RATIO euclidean ward	
		PCA euclidean weighted	
		RATIO euclidean tsvq	
		PCA euclidean average	
	3	MR agree average	0.1446
		MR sim average	
		STATUS sim average	
		STATUS sim diana	
		STATUS agree average	
		MR sim weighted	
		STATUS sim weighted	
	4	MR agree average	0.2418
		STATUS agree average	
		MR sim average	
		STATUS sim average	
		MR agree complete	
		MR sim weighted	
	5	MR agree average	0.0772
		STATUS agree average	
		MR agree complete	
		MR sim weighted	
		MR sim average	
	6	MR agree average	0.2372
		STATUS agree average	
		MR agree complete	
		MR sim average	
		MR sim weighted	
		PCA euclidean average	
	7	MR agree average	1
		MR agree complete	
		MR sim average	
		MR sim weighted	
		STATUS agree average	
	8	MR agree average	0.7509
		MR conc average	
		STATUS agree average	
		MR agree complete	
		MR sim weighted	
		MR sim average	

Table S 66. Stable class discovery methods and associated p-value for data set **gysin**, partitions from 2 to 8 clusters, similarity coefficient Rogers-Tanimoto and similarity threshold $s^0 = 0.85$.

**Rogers-Tanimoto** $s^0 = 0.85$			
**set**	**nb.clusters**		**p-value**
**gysin**			
	9	MR agree average<br>MR agree complete<br>STATUS agree average	1
	10	STATUS agree average<br>MR agree average	0.0970

Table S 67. Stable class discovery methods and associated p-value for data set **gysin**, partitions from 9 to 10 clusters, similarity coefficient Rogers-Tanimoto and similarity threshold $s^0 = 0.85$.

Rogers-Tanimoto $s^0 = 0.90$			
**set**	**nb.clusters**		**p-value**
**gysin**			
	2	MR sim average	0.3146
		STATUS sim diana	
		MR agree average	
		MR sim diana	
		MR sim weighted	
		STATUS sim average	
		MR pearson average	
		RATIO euclidean ward	
		PCA euclidean weighted	
		RATIO euclidean tsvq	
		PCA euclidean average	
	3	MR agree average	0.1446
		MR sim average	
		STATUS sim average	
		STATUS sim diana	
		STATUS agree average	
		MR sim weighted	
		STATUS sim weighted	
	4	MR agree average	0.2418
		STATUS agree average	
		MR sim average	
		STATUS sim average	
		MR agree complete	
		MR sim weighted	
	5	MR agree average	0.0772
		STATUS agree average	
		MR agree complete	
		MR sim weighted	
		MR sim average	
	6	MR agree average	0.2372
		STATUS agree average	
		MR agree complete	
		MR sim average	
		MR sim weighted	
		PCA euclidean average	
	7	MR agree average	1
		MR agree complete	
		MR sim average	
		MR sim weighted	
		STATUS agree average	
	8	MR agree average	0.7509
		MR conc average	
		STATUS agree average	
		MR agree complete	
		MR sim weighted	
		MR sim average	

Table S 68. Stable class discovery methods and associated p-value for data set **gysin**, partitions from 2 to 8 clusters, similarity coefficient Rogers-Tanimoto and similarity threshold $s^0 = 0.90$.

**Rogers-Tanimoto** $s^0 = 0.90$			
**set**	**nb.clusters**		**p-value**
**gysin**			
	9	MR agree average<br>MR agree complete<br>STATUS agree average	1
	10	STATUS agree average<br>MR agree average	0.0970

Table S 69. Stable class discovery methods and associated p-value for data set **gysin**, partitions from 9 to 10 clusters, similarity coefficient Rogers-Tanimoto and similarity threshold $s^0 = 0.90$.

Rogers-Tanimoto $s^0 = 0.95$			
**set**	**nb.clusters**		**p-value**
**gysin**			
	2	MR sim average	0.3146
		STATUS sim diana	
		MR agree average	
		MR sim diana	
		MR sim weighted	
		STATUS sim average	
		MR pearson average	
		RATIO euclidean ward	
		PCA euclidean weighted	
		RATIO euclidean tsvq	
		PCA euclidean average	
	3	MR agree average	0.1446
		MR sim average	
		STATUS sim average	
		STATUS sim diana	
		STATUS agree average	
		MR sim weighted	
		STATUS sim weighted	
	4	MR agree average	0.2418
		STATUS agree average	
		MR sim average	
		STATUS sim average	
		MR agree complete	
		MR sim weighted	
	5	MR agree average	0.0772
		STATUS agree average	
		MR agree complete	
		MR sim weighted	
		MR sim average	
	6	MR agree average	0.2372
		STATUS agree average	
		MR agree complete	
		MR sim average	
		MR sim weighted	
		PCA euclidean average	
	7	MR agree average	1
		MR agree complete	
		MR sim average	
		MR sim weighted	
		STATUS agree average	
	8	MR agree average	0.7509
		MR conc average	
		STATUS agree average	
		MR agree complete	
		MR sim weighted	
		MR sim average	

Table S 70. Stable class discovery methods and associated p-value for data set **gysin**, partitions from 2 to 8 clusters, similarity coefficient Rogers-Tanimoto and similarity threshold $s^0 = 0.95$.

**Rogers-Tanimoto** $s^0 = 0.95$			
**set**	**nb.clusters**		**p-value**
**gysin**			
	9	MR agree average<br>MR agree complete<br>STATUS agree average	1
	10	STATUS agree average<br>MR agree average	0.0970

Table S 71. Stable class discovery methods and associated p-value for data set **gysin**, partitions from 9 to 10 clusters, similarity coefficient Rogers-Tanimoto and similarity threshold $s^0 = 0.95$.

**Rogers-Tanimoto** $s^0 = 0.97$			
**set**	**nb.clusters**		**p-value**
**gysin**			
	2	MR sim average	0.3146
		STATUS sim diana	
		MR agree average	
		MR sim diana	
		MR sim weighted	
		STATUS sim average	
		MR pearson average	
		RATIO euclidean ward	
		PCA euclidean weighted	
		RATIO euclidean tsvq	
		PCA euclidean average	
	3	MR agree average	0.1446
		MR sim average	
		STATUS sim average	
		STATUS sim diana	
		STATUS agree average	
		MR sim weighted	
		STATUS sim weighted	
	4	MR agree average	0.2418
		STATUS agree average	
		MR sim average	
		STATUS sim average	
		MR agree complete	
		MR sim weighted	
	5	MR agree average	0.0772
		STATUS agree average	
		MR agree complete	
		MR sim weighted	
		MR sim average	
	6	MR agree average	0.2372
		STATUS agree average	
		MR agree complete	
		MR sim average	
		MR sim weighted	
		PCA euclidean average	
	7	MR agree average	1
		MR agree complete	
		MR sim average	
		MR sim weighted	
		STATUS agree average	
	8	MR agree average	0.7509
		MR conc average	
		STATUS agree average	
		MR agree complete	
		MR sim weighted	
		MR sim average	

Table S 72. Stable class discovery methods and associated p-value for data set **gysin**, partitions from 2 to 8 clusters, similarity coefficient Rogers-Tanimoto and similarity threshold $s^0 = 0.97$.

**Rogers-Tanimoto** $s^0 = 0.97$			
**set**	**nb.clusters**		**p-value**
**gysin**			
	9	MR agree average<br>MR agree complete<br>STATUS agree average	1
	10	STATUS agree average<br>MR agree average	0.0970

Table S 73. Stable class discovery methods and associated p-value for data set **gysin**, partitions from 9 to 10 clusters, similarity coefficient Rogers-Tanimoto and similarity threshold $s^0 = 0.97$.

Rogers-Tanimoto $s^0 = 0.99$			
**set**	**nb.clusters**		**p-value**
**gysin**			
	2	MR sim average	0.3146
		STATUS sim diana	
		MR agree average	
		MR sim diana	
		MR sim weighted	
		STATUS sim average	
		MR pearson average	
		RATIO euclidean ward	
		PCA euclidean weighted	
		RATIO euclidean tsvq	
		PCA euclidean average	
	3	MR agree average	0.1446
		MR sim average	
		STATUS sim average	
		STATUS sim diana	
		STATUS agree average	
		MR sim weighted	
		STATUS sim weighted	
	4	MR agree average	0.2418
		STATUS agree average	
		MR sim average	
		STATUS sim average	
		MR agree complete	
		MR sim weighted	
	5	MR agree average	0.0772
		STATUS agree average	
		MR agree complete	
		MR sim weighted	
		MR sim average	
	6	MR agree average	0.2372
		STATUS agree average	
		MR agree complete	
		MR sim average	
		MR sim weighted	
		PCA euclidean average	
	7	MR agree average	1
		MR agree complete	
		MR sim average	
		MR sim weighted	
		STATUS agree average	
	8	MR agree average	0.7509
		MR conc average	
		STATUS agree average	
		MR agree complete	
		MR sim weighted	
		MR sim average	

Table S 74. Stable class discovery methods and associated p-value for data set **gysin**, partitions from 2 to 8 clusters, similarity coefficient Rogers-Tanimoto and similarity threshold $s^0 = 0.99$.

**Rogers-Tanimoto** $s^0 = 0.99$			
**set**	**nb.clusters**		**p-value**
**gysin**			
	9	MR agree average<br>MR agree complete<br>STATUS agree average	1
	10	STATUS agree average<br>MR agree average	0.0970

Table S 75. Stable class discovery methods and associated p-value for data set **gysin**, partitions from 9 to 10 clusters, similarity coefficient Rogers-Tanimoto and similarity threshold $s^0 = 0.99$.

**Jaccard** $s^0 = 0.85$			
**set**	**nb.clusters**		**p-value**
**blaveri**			
	2	MR pearson average<br>MR pearson kcentroids<br>MR pearson ward<br>MR pearson weighted	0.1462
	3	MR sim average<br>MR sim weighted	0.3604
	4	MR sim average<br>MR sim weighted	0.7027
	5	MR sim average<br>MR sim weighted	1
	6	MR sim average<br>MR sim weighted	0.5166
	7	MR sim average<br>MR sim weighted	1
	8	MR sim average<br>MR sim weighted	1
	9	MR sim average	1
	10	MR sim average	0.2978

Table S 76. Stable class discovery methods and associated p-value for data set **blaveri**, partitions from 2 to 10 clusters, similarity coefficient Jaccard and similarity threshold $s^0 = 0.85$.

**Jaccard** $s^0 = 0.90$			
**set**	**nb.clusters**		**p-value**
**blaveri**			
	2	MR pearson average<br>MR pearson kcentroids<br>MR pearson ward<br>MR pearson weighted	0.1462
	3	MR sim average<br>MR sim weighted	0.3604
	4	MR sim average<br>MR sim weighted	0.7027
	5	MR sim average<br>MR sim weighted	1
	6	MR sim average<br>MR sim weighted	0.5166
	7	MR sim average<br>MR sim weighted	1
	8	MR sim average<br>MR sim weighted	1
	9	MR sim average	1
	10	MR sim average	0.2978

Table S 77. Stable class discovery methods and associated p-value for data set **blaveri**, partitions from 2 to 10 clusters, similarity coefficient Jaccard and similarity threshold $s^0 = 090$.

**Jaccard** $s^0 = 0.95$			
**set**	**nb.clusters**		**p-value**
**blaveri**			
	2	MR pearson average<br>MR pearson kcentroids<br>MR pearson ward<br>MR pearson weighted	0.1462
	3	MR sim average<br>MR sim weighted	0.3604
	4	MR sim average<br>MR sim weighted	0.7027
	5	MR sim average<br>MR sim weighted	1
	6	MR sim average<br>MR sim weighted	0.5166
	7	MR sim average<br>MR sim weighted	1
	8	MR sim average<br>MR sim weighted	1
	9	MR sim average	1
	10	MR sim average	0.2978

Table S 78. Stable class discovery methods and associated *p*-value for data set **blaveri**, partitions from 2 to 10 clusters, similarity coefficient Jaccard and similarity threshold $s^0 = 0.95$.

**Jaccard** $s^0 = 0.97$			
**set**	**nb.clusters**		**p-value**
**blaveri**			
	2	MR pearson average<br>MR pearson kcentroids<br>MR pearson ward<br>MR pearson weighted	0.1462
	3	MR sim average<br>MR sim weighted	0.3604
	4	MR sim average<br>MR sim weighted	0.7027
	5	MR sim average<br>MR sim weighted	1
	6	MR sim average<br>MR sim weighted	0.5166
	7	MR sim average<br>MR sim weighted	1
	8	MR sim average<br>MR sim weighted	1
	9	MR sim average	1
	10	MR sim average	0.2978

Table S 79. Stable class discovery methods and associated *p*-value for data set **blaveri**, partitions from 2 to 10 clusters, similarity coefficient Jaccard and similarity threshold $s^0 = 0.97$.

Jaccard $s^0 = 0.99$			
**set**	**nb.clusters**		**p-value**
**blaveri**			
	2	MR pearson average<br>MR pearson kcentroids<br>MR pearson ward<br>MR pearson weighted	0.1462
	3	MR sim average<br>MR sim weighted	0.3604
	4	MR sim average<br>MR sim weighted	0.7027
	5	MR sim average<br>MR sim weighted	1
	6	MR sim average<br>MR sim weighted	0.5166
	7	MR sim average<br>MR sim weighted	1
	8	MR sim average<br>MR sim weighted	1
	9	MR sim average	1
	10	MR sim average	0.2978

Table S 80. Stable class discovery methods and associated p-value for data set **blaveri**, partitions from 2 to 10 clusters, similarity coefficient Jaccard and similarity threshold $s^0 = 0.99$.

Simple Matching $s^0 = 0.85$			
**set**	**nb.clusters**		**p-value**
**blaveri**			
	2	MR pearson average<br>MR pearson kcentroids<br>MR pearson ward<br>MR pearson weighted	0.1462
	3	MR sim average<br>MR sim weighted	0.3604
	4	MR sim average<br>MR sim weighted	0.7027
	5	MR sim average<br>MR sim weighted	1
	6	MR sim average<br>MR sim weighted	0.5166
	7	MR sim average<br>MR sim weighted	1
	8	MR sim average<br>MR sim weighted	1
	9	MR sim average	1
	10	MR sim average	0.2978

Table S 81. Stable class discovery methods and associated p-value for data set **blaveri**, partitions from 2 to 10 clusters, similarity coefficient Simple Matching and similarity threshold $s^0 = 0.85$.

Simple Matching $s^0 = 0.90$			
**set**	**nb.clusters**		**p-value**
**blaveri**			
	2	MR pearson average	0.1462
		MR pearson kcentroids	
		MR pearson ward	
		MR pearson weighted	
	3	MR sim average	0.3604
		MR sim weighted	
	4	MR sim average	0.7027
		MR sim weighted	
	5	MR sim average	1
		MR sim weighted	
	6	MR sim average	0.5166
		MR sim weighted	
	7	MR sim average	1
		MR sim weighted	
	8	MR sim average	1
		MR sim weighted	
	9	MR sim average	1
	10	MR sim average	0.2978

Table S 82. Stable class discovery methods and associated p-value for data set **blaveri**, partitions from 2 to 10 clusters, similarity coefficient Simple Matching and similarity threshold $s^0 = 0.90$.

Simple Matching $s^0 = 0.95$			
**set**	**nb.clusters**		**p-value**
**blaveri**			
	2	MR pearson average	0.1462
		MR pearson kcentroids	
		MR pearson ward	
		MR pearson weighted	
	3	MR sim average	0.3604
		MR sim weighted	
	4	MR sim average	0.7027
		MR sim weighted	
	5	MR sim average	1
		MR sim weighted	
	6	MR sim average	0.5166
		MR sim weighted	
	7	MR sim average	1
		MR sim weighted	
	8	MR sim average	1
		MR sim weighted	
	9	MR sim average	1
	10	MR sim average	0.2978

Table S 83. Stable class discovery methods and associated p-value for data set **blaveri**, partitions from 2 to 10 clusters, similarity coefficient Simple Matching and similarity threshold $s^0 = 0.95$.

**Simple Matching** $s^0 = 0.97$			
**set**	**nb.clusters**		**p-value**
**blaveri**			
	2	MR pearson average<br>MR pearson kcentroids<br>MR pearson ward<br>MR pearson weighted	0.1462
	3	MR sim average<br>MR sim weighted	0.3604
	4	MR sim average<br>MR sim weighted	0.7027
	5	MR sim average<br>MR sim weighted	1
	6	MR sim average<br>MR sim weighted	0.5166
	7	MR sim average<br>MR sim weighted	1
	8	MR sim average<br>MR sim weighted	1
	9	MR sim average	1
	10	MR sim average	0.2978

Table S 84. Stable class discovery methods and associated p-value for data set **blaveri**, partitions from 2 to 10 clusters, similarity coefficient Simple Matching and similarity threshold $s^0 = 0.97$.

**Simple Matching** $s^0 = 0.99$			
**set**	**nb.clusters**		**p-value**
**blaveri**			
	2	MR pearson average<br>MR pearson kcentroids<br>MR pearson ward<br>MR pearson weighted	0.1462
	3	MR sim average<br>MR sim weighted	0.3604
	4	MR sim average<br>MR sim weighted	0.7027
	5	MR sim average<br>MR sim weighted	1
	6	MR sim average<br>MR sim weighted	0.5166
	7	MR sim average<br>MR sim weighted	1
	8	MR sim average<br>MR sim weighted	1
	9	MR sim average	1
	10	MR sim average	0.2978

Table S 85. Stable class discovery methods and associated p-value for data set **blaveri**, partitions from 2 to 10 clusters, similarity coefficient Simple Matching and similarity threshold $s^0 = 0.99$.

**Rogers-Tanimoto** $s^0 = 0.85$			
**set**	**nb.clusters**		**p-value**
**blaveri**			
	2	MR pearson average<br>MR pearson kcentroids<br>MR pearson ward<br>MR pearson weighted	0.1462
	3	MR sim average<br>MR sim weighted	0.3604
	4	MR sim average<br>MR sim weighted	0.7027
	5	MR sim average<br>MR sim weighted	1
	6	MR sim average<br>MR sim weighted	0.5166
	7	MR sim average<br>MR sim weighted	1
	8	MR sim average<br>MR sim weighted	1
	9	MR sim average	1
	10	MR sim average	0.2978

Table S 86. Stable class discovery methods and associated *p*-value for data set **blaveri**, partitions from 2 to 10 clusters, similarity coefficient Rogers-Tanimoto and similarity threshold $s^0 = 0.85$.

**Rogers-Tanimoto** $s^0 = 0.90$			
**set**	**nb.clusters**		**p-value**
**blaveri**			
	2	MR pearson average<br>MR pearson kcentroids<br>MR pearson ward<br>MR pearson weighted	0.1462
	3	MR sim average<br>MR sim weighted	0.3604
	4	MR sim average<br>MR sim weighted	0.7027
	5	MR sim average<br>MR sim weighted	1
	6	MR sim average<br>MR sim weighted	0.5166
	7	MR sim average<br>MR sim weighted	1
	8	MR sim average<br>MR sim weighted	1
	9	MR sim average	1
	10	MR sim average	0.2978

Table S 87. Stable class discovery methods and associated *p*-value for data set **blaveri**, partitions from 2 to 10 clusters, similarity coefficient Rogers-Tanimoto and similarity threshold $s^0 = 0.90$.

**Rogers-Tanimoto** $s^0 = 0.95$			
**set**	**nb.clusters**		**p-value**
**blaveri**			
	2	MR pearson average	0.1462
		MR pearson kcentroids	
		MR pearson ward	
		MR pearson weighted	
	3	MR sim average	0.3604
		MR sim weighted	
	4	MR sim average	0.7027
		MR sim weighted	
	5	MR sim average	1
		MR sim weighted	
	6	MR sim average	0.5166
		MR sim weighted	
	7	MR sim average	1
		MR sim weighted	
	8	MR sim average	1
		MR sim weighted	
	9	MR sim average	1
	10	MR sim average	0.2978

Table S 88. Stable class discovery methods and associated p-value for data set **blaveri**, partitions from 2 to 10 clusters, similarity coefficient Rogers-Tanimoto and similarity threshold $s^0 = 0.95$.

**Rogers-Tanimoto** $s^0 = 0.97$			
**set**	**nb.clusters**		**p-value**
**blaveri**			
	2	MR pearson average	0.1462
		MR pearson kcentroids	
		MR pearson ward	
		MR pearson weighted	
	3	MR sim average	0.3604
		MR sim weighted	
	4	MR sim average	0.7027
		MR sim weighted	
	5	MR sim average	1
		MR sim weighted	
	6	MR sim average	0.5166
		MR sim weighted	
	7	MR sim average	1
		MR sim weighted	
	8	MR sim average	1
		MR sim weighted	
	9	MR sim average	1
	10	MR sim average	0.2978

Table S 89. Stable class discovery methods and associated p-value for data set **blaveri**, partitions from 2 to 10 clusters, similarity coefficient Rogers-Tanimoto and similarity threshold $s^0 = 0.97$.

Rogers-Tanimoto $s^0 = 0.99$			
**set**	**nb.clusters**		**p-value**
**blaveri**			
	2	MR pearson average<br>MR pearson kcentroids<br>MR pearson ward<br>MR pearson weighted	0.1462
	3	MR sim average<br>MR sim weighted	0.3604
	4	MR sim average<br>MR sim weighted	0.7027
	5	MR sim average<br>MR sim weighted	1
	6	MR sim average<br>MR sim weighted	0.5166
	7	MR sim average<br>MR sim weighted	1
	8	MR sim average<br>MR sim weighted	1
	9	MR sim average	1
	10	MR sim average	0.2978

Table S 90. Stable class discovery methods and associated p-value for data set **blaveri**, partitions from 2 to 10 clusters, similarity coefficient Rogers-Tanimoto and similarity threshold $s^0 = 0.99$.

**Jaccard** $s^0 = 0.85$ **set**	**nb.clusters**		**p-value**
**patil**			
	2	MR pearson average	0.4056
		MR pearson weighted	
		MR sim weighted	
		MR sim average	
	3	MR sim average	0.0609
		STATUS agree average	
		MR agree average	
		STATUS sim average	
		MR pearson average	
		STATUS manhattan average	
		RATIO manhattan average	
		SMOOTH manhattan average	
		STATUS sim diana	
		MR sim weighted	
		RATIO euclidean average	
		SMOOTH euclidean average	
		PCA euclidean average	
	4	PCA euclidean average	0.6660
		SMOOTH euclidean average	
		STATUS euclidean average	
		MR sim diana	

Table S 91. Stable class discovery methods and associated p-value for data set **patil**, partitions from 2 to 4 clusters, similarity coefficient Jaccard and similarity threshold $s^0 = 0.85$.

**Jaccard** $s^0 = 0.85$ **set**	**nb.clusters**		**p-value**
**patil**			
	5	STATUS agree average	0.0771
		PCA euclidean average	
		MR sim average	
		MR agree average	
		STATUS sim average	
		SMOOTH euclidean average	
		RATIO euclidean average	
		SMOOTH euclidean diana	
		MR sim diana	
		PCA euclidean diana	
	6	RATIO euclidean average	0.4908
		STATUS agree average	
		STATUS sim average	
		MR sim average	
		SMOOTH euclidean average	
		MR agree average	
		RATIO euclidean diana	
		PCA euclidean average	
	7	MR agree average	0.0900
		SMOOTH euclidean average	
		STATUS agree average	
		RATIO euclidean average	
		MR sim average	
		RATIO euclidean diana	
	8	RATIO euclidean average	0.0931
		SMOOTH euclidean average	
		MR agree average	
		MR sim average	
		PCA manhattan average	
		PCA euclidean average	
		SMOOTH euclidean diana	
		STATUS agree average	
	9	RATIO euclidean average	0.0513
		MR agree average	
		MR sim average	
	10	MR agree average	1

Table S 92. Stable class discovery methods and associated p-value for data set **patil**, partitions from 5 to 10 clusters, similarity coefficient Jaccard and similarity threshold $s^0 = 0.85$.

Jaccard $s^0 = 0.90$ set	nb.clusters		p-value
**patil**			
	2	MR pearson average	0.4056
		MR pearson weighted	
		MR sim weighted	
		MR sim average	
	3	MR sim average	0.0609
		STATUS agree average	
		MR agree average	
		STATUS sim average	
		MR pearson average	
		STATUS manhattan average	
		RATIO manhattan average	
		SMOOTH manhattan average	
		STATUS sim diana	
		MR sim weighted	
		RATIO euclidean average	
		SMOOTH euclidean average	
		PCA euclidean average	
	4	PCA euclidean average	0.6660
		SMOOTH euclidean average	
		STATUS euclidean average	
		MR sim diana	

Table S 93. Stable class discovery methods and associated *p*-value for data set **patil**, partitions from 2 to 4 clusters, similarity coefficient Jaccard and similarity threshold $s^0 = 0.90$.

Jaccard $s^0 = 0.90$ set	nb.clusters		p-value
**patil**			
	5	STATUS agree average	0.0771
		PCA euclidean average	
		MR sim average	
		MR agree average	
		STATUS sim average	
		SMOOTH euclidean average	
		RATIO euclidean average	
		SMOOTH euclidean diana	
		MR sim diana	
		PCA euclidean diana	
	6	RATIO euclidean average	0.4908
		STATUS agree average	
		STATUS sim average	
		MR sim average	
		SMOOTH euclidean average	
		MR agree average	
		RATIO euclidean diana	
		PCA euclidean average	
	7	MR agree average	0.0900
		SMOOTH euclidean average	
		STATUS agree average	
		RATIO euclidean average	
		MR sim average	
		RATIO euclidean diana	
	8	RATIO euclidean average	0.0931
		SMOOTH euclidean average	
		MR agree average	
		MR sim average	
		PCA manhattan average	
		PCA euclidean average	
		SMOOTH euclidean diana	
		STATUS agree average	
	9	RATIO euclidean average	0.0513
		MR agree average	
		MR sim average	
	10	MR agree average	1

Table S 94. Stable class discovery methods and associated p-value for data set **patil**, partitions from 5 to 10 clusters, similarity coefficient Jaccard and similarity threshold $s^0 = 0.90$.

**Jaccard** $s^0 = 0.95$ **set**	**nb.clusters**		**p-value**
**patil**			
	2	MR pearson average	0.4056
		MR pearson weighted	
		MR sim weighted	
		MR sim average	
	3	MR sim average	0.0609
		STATUS agree average	
		MR agree average	
		STATUS sim average	
		MR pearson average	
		STATUS manhattan average	
		RATIO manhattan average	
		SMOOTH manhattan average	
		STATUS sim diana	
		MR sim weighted	
		RATIO euclidean average	
		SMOOTH euclidean average	
		PCA euclidean average	
	4	PCA euclidean average	0.6660
		SMOOTH euclidean average	
		STATUS euclidean average	
		MR sim diana	

Table S 95. Stable class discovery methods and associated p-value for data set **patil**, partitions from 2 to 4 clusters, similarity coefficient Jaccard and similarity threshold $s^0 = 0.95$.

Jaccard $s^0 = 0.95$ set	nb.clusters		p-value
**patil**			
	5	STATUS agree average	0.0771
		PCA euclidean average	
		MR sim average	
		MR agree average	
		STATUS sim average	
		SMOOTH euclidean average	
		RATIO euclidean average	
		SMOOTH euclidean diana	
		MR sim diana	
		PCA euclidean diana	
	6	RATIO euclidean average	0.4908
		STATUS agree average	
		STATUS sim average	
		MR sim average	
		SMOOTH euclidean average	
		MR agree average	
		RATIO euclidean diana	
		PCA euclidean average	
	7	MR agree average	0.0900
		SMOOTH euclidean average	
		STATUS agree average	
		RATIO euclidean average	
		MR sim average	
		RATIO euclidean diana	
	8	RATIO euclidean average	0.0931
		SMOOTH euclidean average	
		MR agree average	
		MR sim average	
		PCA manhattan average	
		PCA euclidean average	
		SMOOTH euclidean diana	
		STATUS agree average	
	9	RATIO euclidean average	0.0513
		MR agree average	
		MR sim average	
	10	MR agree average	1

Table S 96. Stable class discovery methods and associated p-value for data set **patil**, partitions from 5 to 10 clusters, similarity coefficient Jaccard and similarity threshold $s^0 = 0.95$.

**Jaccard** $s^0 = 0.97$ **set**	**nb.clusters**		**p-value**
**patil**			
	2	MR pearson average	0.4056
		MR pearson weighted	
		MR sim weighted	
		MR sim average	
	3	MR sim average	0.0609
		STATUS agree average	
		MR agree average	
		STATUS sim average	
		MR pearson average	
		STATUS manhattan average	
		RATIO manhattan average	
		SMOOTH manhattan average	
		STATUS sim diana	
		MR sim weighted	
		RATIO euclidean average	
		SMOOTH euclidean average	
		PCA euclidean average	
	4	PCA euclidean average	0.6660
		SMOOTH euclidean average	
		STATUS euclidean average	
		MR sim diana	

Table S 97. Stable class discovery methods and associated p-value for data set **patil**, partitions from 2 to 4 clusters, similarity coefficient Jaccard and similarity threshold $s^0 = 0.97$.

**Jaccard** $s^0 = 0.97$ **set**	**nb.clusters**		**p-value**
**patil**			
	5	STATUS agree average	0.0771
		PCA euclidean average	
		MR sim average	
		MR agree average	
		STATUS sim average	
		SMOOTH euclidean average	
		RATIO euclidean average	
		SMOOTH euclidean diana	
		MR sim diana	
		PCA euclidean diana	
	6	RATIO euclidean average	0.4908
		STATUS agree average	
		STATUS sim average	
		MR sim average	
		SMOOTH euclidean average	
		MR agree average	
		RATIO euclidean diana	
		PCA euclidean average	
	7	MR agree average	0.0900
		SMOOTH euclidean average	
		STATUS agree average	
		RATIO euclidean average	
		MR sim average	
		RATIO euclidean diana	
	8	RATIO euclidean average	0.0931
		SMOOTH euclidean average	
		MR agree average	
		MR sim average	
		PCA manhattan average	
		PCA euclidean average	
		SMOOTH euclidean diana	
		STATUS agree average	
	9	RATIO euclidean average	0.0513
		MR agree average	
		MR sim average	
	10	MR agree average	1

Table S 98. Stable class discovery methods and associated p-value for data set **patil**, partitions from 5 to 10 clusters, similarity coefficient Jaccard and similarity threshold $s^0 = 0.97$.

Jaccard $s^0 = 0.99$ set	nb.clusters		p-value
**patil**			
	2	MR pearson average	0.4056
		MR pearson weighted	
		MR sim weighted	
		MR sim average	
	3	MR sim average	0.0609
		STATUS agree average	
		MR agree average	
		STATUS sim average	
		MR pearson average	
		STATUS manhattan average	
		RATIO manhattan average	
		SMOOTH manhattan average	
		STATUS sim diana	
		MR sim weighted	
		RATIO euclidean average	
		SMOOTH euclidean average	
		PCA euclidean average	
	4	PCA euclidean average	0.6660
		SMOOTH euclidean average	
		STATUS euclidean average	
		MR sim diana	

Table S 99. Stable class discovery methods and associated p-value for data set **patil**, partitions from 2 to 4 clusters, similarity coefficient Jaccard and similarity threshold $s^0 = 0.99$.

Jaccard $s^0 = 0.99$ set	nb.clusters		p-value
**patil**			
	5	STATUS agree average	0.0771
		PCA euclidean average	
		MR sim average	
		MR agree average	
		STATUS sim average	
		SMOOTH euclidean average	
		RATIO euclidean average	
		SMOOTH euclidean diana	
		MR sim diana	
		PCA euclidean diana	
	6	RATIO euclidean average	0.4908
		STATUS agree average	
		STATUS sim average	
		MR sim average	
		SMOOTH euclidean average	
		MR agree average	
		RATIO euclidean diana	
		PCA euclidean average	
	7	MR agree average	0.0900
		SMOOTH euclidean average	
		STATUS agree average	
		RATIO euclidean average	
		MR sim average	
		RATIO euclidean diana	
	8	RATIO euclidean average	0.0931
		SMOOTH euclidean average	
		MR agree average	
		MR sim average	
		PCA manhattan average	
		PCA euclidean average	
		SMOOTH euclidean diana	
		STATUS agree average	
	9	RATIO euclidean average	0.0513
		MR agree average	
		MR sim average	
	10	MR agree average	1

Table S 100. Stable class discovery methods and associated p-value for data set **patil**, partitions from 5 to 10 clusters, similarity coefficient Jaccard and similarity threshold $s^0 = 0.99$.

**Single Matching $s^0 = 0.85$ set**	**nb.clusters**		**p-value**
**patil**			
	2	MR pearson average	0.4056
		MR pearson weighted	
		MR sim weighted	
		MR sim average	
	3	MR sim average	0.0609
		STATUS agree average	
		MR agree average	
		STATUS sim average	
		MR pearson average	
		STATUS manhattan average	
		RATIO manhattan average	
		SMOOTH manhattan average	
		STATUS sim diana	
		MR sim weighted	
		RATIO euclidean average	
		SMOOTH euclidean average	
		PCA euclidean average	
	4	PCA euclidean average	0.6660
		SMOOTH euclidean average	
		STATUS euclidean average	
		MR sim diana	

Table S 101. Stable class discovery methods and associated p-value for data set **patil**, partitions from 2 to 4 clusters, similarity coefficient Single Matching and similarity threshold $s^0 = 0.85$.

Single Matching $s^0 = 0.85$ set	nb.clusters		p-value
**patil**			
	5	STATUS agree average	0.0771
		PCA euclidean average	
		MR sim average	
		MR agree average	
		STATUS sim average	
		SMOOTH euclidean average	
		RATIO euclidean average	
		SMOOTH euclidean diana	
		MR sim diana	
		PCA euclidean diana	
	6	RATIO euclidean average	0.4908
		STATUS agree average	
		STATUS sim average	
		MR sim average	
		SMOOTH euclidean average	
		MR agree average	
		RATIO euclidean diana	
		PCA euclidean average	
	7	MR agree average	0.0900
		SMOOTH euclidean average	
		STATUS agree average	
		RATIO euclidean average	
		MR sim average	
		RATIO euclidean diana	
	8	RATIO euclidean average	0.0931
		SMOOTH euclidean average	
		MR agree average	
		MR sim average	
		PCA manhattan average	
		PCA euclidean average	
		SMOOTH euclidean diana	
		STATUS agree average	
	9	RATIO euclidean average	0.0513
		MR agree average	
		MR sim average	
	10	MR agree average	1

Table S 102. Stable class discovery methods and associated p-value for data set **patil**, partitions from 5 to 10 clusters, similarity coefficient Single Matching and similarity threshold $s^0 = 0.85$.

**Single Matching** $s^0 = 0.90$ **set**	**nb.clusters**		**p-value**
**patil**			
	2	MR pearson average	0.4056
		MR pearson weighted	
		MR sim weighted	
		MR sim average	
	3	MR sim average	0.0609
		STATUS agree average	
		MR agree average	
		STATUS sim average	
		MR pearson average	
		STATUS manhattan average	
		RATIO manhattan average	
		SMOOTH manhattan average	
		STATUS sim diana	
		MR sim weighted	
		RATIO euclidean average	
		SMOOTH euclidean average	
		PCA euclidean average	
	4	PCA euclidean average	0.6660
		SMOOTH euclidean average	
		STATUS euclidean average	
		MR sim diana	

Table S 103. Stable class discovery methods and associated p-value for data set **patil**, partitions from 2 to 4 clusters, similarity coefficient Single Matching and similarity threshold $s^0 = 0.90$.

Single Matching $s^0 = 0.90$ set	nb.clusters		p-value
**patil**			
	5	STATUS agree average	0.0771
		PCA euclidean average	
		MR sim average	
		MR agree average	
		STATUS sim average	
		SMOOTH euclidean average	
		RATIO euclidean average	
		SMOOTH euclidean diana	
		MR sim diana	
		PCA euclidean diana	
	6	RATIO euclidean average	0.4908
		STATUS agree average	
		STATUS sim average	
		MR sim average	
		SMOOTH euclidean average	
		MR agree average	
		RATIO euclidean diana	
		PCA euclidean average	
	7	MR agree average	0.0900
		SMOOTH euclidean average	
		STATUS agree average	
		RATIO euclidean average	
		MR sim average	
		RATIO euclidean diana	
	8	RATIO euclidean average	0.0931
		SMOOTH euclidean average	
		MR agree average	
		MR sim average	
		PCA manhattan average	
		PCA euclidean average	
		SMOOTH euclidean diana	
		STATUS agree average	
	9	RATIO euclidean average	0.0513
		MR agree average	
		MR sim average	
	10	MR agree average	1

Table S 104. Stable class discovery methods and associated p-value for data set **patil**, partitions from 5 to 10 clusters, similarity coefficient Single Matching and similarity threshold $s^0 = 0.90$.

**Single Matching** $s^0 = 0.95$ **set**	**nb.clusters**		**p-value**
**patil**			
	2	MR pearson average	0.4056
		MR pearson weighted	
		MR sim weighted	
		MR sim average	
	3	MR sim average	0.0609
		STATUS agree average	
		MR agree average	
		STATUS sim average	
		MR pearson average	
		STATUS manhattan average	
		RATIO manhattan average	
		SMOOTH manhattan average	
		STATUS sim diana	
		MR sim weighted	
		RATIO euclidean average	
		SMOOTH euclidean average	
		PCA euclidean average	
	4	PCA euclidean average	0.6660
		SMOOTH euclidean average	
		STATUS euclidean average	
		MR sim diana	

Table S 105. Stable class discovery methods and associated p-value for data set **patil**, partitions from 2 to 4 clusters, similarity coefficient Single Matching and similarity threshold $s^0 = 0.95$.

Single Matching $s^0 = 0.95$ set	nb.clusters		p-value
**patil**			
	5	STATUS agree average	0.0771
		PCA euclidean average	
		MR sim average	
		MR agree average	
		STATUS sim average	
		SMOOTH euclidean average	
		RATIO euclidean average	
		SMOOTH euclidean diana	
		MR sim diana	
		PCA euclidean diana	
	6	RATIO euclidean average	0.4908
		STATUS agree average	
		STATUS sim average	
		MR sim average	
		SMOOTH euclidean average	
		MR agree average	
		RATIO euclidean diana	
		PCA euclidean average	
	7	MR agree average	0.0900
		SMOOTH euclidean average	
		STATUS agree average	
		RATIO euclidean average	
		MR sim average	
		RATIO euclidean diana	
	8	RATIO euclidean average	0.0931
		SMOOTH euclidean average	
		MR agree average	
		MR sim average	
		PCA manhattan average	
		PCA euclidean average	
		SMOOTH euclidean diana	
		STATUS agree average	
	9	RATIO euclidean average	0.0513
		MR agree average	
		MR sim average	
	10	MR agree average	1

Table S 106. Stable class discovery methods and associated p-value for data set **patil**, partitions from 5 to 10 clusters, similarity coefficient Single Matching and similarity threshold $s^0 = 0.95$.

Single Matching $s^0 = 0.97$ set	nb.clusters		p-value
**patil**			
	2	MR pearson average	0.4056
		MR pearson weighted	
		MR sim weighted	
		MR sim average	
	3	MR sim average	0.0609
		STATUS agree average	
		MR agree average	
		STATUS sim average	
		MR pearson average	
		STATUS manhattan average	
		RATIO manhattan average	
		SMOOTH manhattan average	
		STATUS sim diana	
		MR sim weighted	
		RATIO euclidean average	
		SMOOTH euclidean average	
		PCA euclidean average	
	4	PCA euclidean average	0.6660
		SMOOTH euclidean average	
		STATUS euclidean average	
		MR sim diana	

Table S 107. Stable class discovery methods and associated p-value for data set **patil**, partitions from 2 to 4 clusters, similarity coefficient Single Matching and similarity threshold $s^0 = 0.97$.

Single Matching $s^0 = 0.97$ set	nb.clusters		p-value
**patil**			
	5	STATUS agree average	0.0771
		PCA euclidean average	
		MR sim average	
		MR agree average	
		STATUS sim average	
		SMOOTH euclidean average	
		RATIO euclidean average	
		SMOOTH euclidean diana	
		MR sim diana	
		PCA euclidean diana	
	6	RATIO euclidean average	0.4908
		STATUS agree average	
		STATUS sim average	
		MR sim average	
		SMOOTH euclidean average	
		MR agree average	
		RATIO euclidean diana	
		PCA euclidean average	
	7	MR agree average	0.0900
		SMOOTH euclidean average	
		STATUS agree average	
		RATIO euclidean average	
		MR sim average	
		RATIO euclidean diana	
	8	RATIO euclidean average	0.0931
		SMOOTH euclidean average	
		MR agree average	
		MR sim average	
		PCA manhattan average	
		PCA euclidean average	
		SMOOTH euclidean diana	
		STATUS agree average	
	9	RATIO euclidean average	0.0513
		MR agree average	
		MR sim average	
	10	MR agree average	1

Table S 108. Stable class discovery methods and associated p-value for data set **patil**, partitions from 5 to 10 clusters, similarity coefficient Single Matching and similarity threshold $s^0 = 0.97$.

**Single Matching** $s^0 = 0.99$ **set**	**nb.clusters**		**p-value**
**patil**			
	2	MR pearson average	0.4056
		MR pearson weighted	
		MR sim weighted	
		MR sim average	
	3	MR sim average	0.0609
		STATUS agree average	
		MR agree average	
		STATUS sim average	
		MR pearson average	
		STATUS manhattan average	
		RATIO manhattan average	
		SMOOTH manhattan average	
		STATUS sim diana	
		MR sim weighted	
		RATIO euclidean average	
		SMOOTH euclidean average	
		PCA euclidean average	
	4	PCA euclidean average	0.6660
		SMOOTH euclidean average	
		STATUS euclidean average	
		MR sim diana	

Table S 109. Stable class discovery methods and associated p-value for data set **patil**, partitions from 2 to 4 clusters, similarity coefficient Single Matching and similarity threshold $s^0 = 0.99$.

**Single Matching** $s^0 = 0.99$ **set**	**nb.clusters**		**p-value**
**patil**			
	5	STATUS agree average	0.0771
		PCA euclidean average	
		MR sim average	
		MR agree average	
		STATUS sim average	
		SMOOTH euclidean average	
		RATIO euclidean average	
		SMOOTH euclidean diana	
		MR sim diana	
		PCA euclidean diana	
	6	RATIO euclidean average	0.4908
		STATUS agree average	
		STATUS sim average	
		MR sim average	
		SMOOTH euclidean average	
		MR agree average	
		RATIO euclidean diana	
		PCA euclidean average	
	7	MR agree average	0.0900
		SMOOTH euclidean average	
		STATUS agree average	
		RATIO euclidean average	
		MR sim average	
		RATIO euclidean diana	
	8	RATIO euclidean average	0.0931
		SMOOTH euclidean average	
		MR agree average	
		MR sim average	
		PCA manhattan average	
		PCA euclidean average	
		SMOOTH euclidean diana	
		STATUS agree average	
	9	RATIO euclidean average	0.0513
		MR agree average	
		MR sim average	
	10	MR agree average	1

Table S 110. Stable class discovery methods and associated p-value for data set **patil**, partitions from 5 to 10 clusters, similarity coefficient Single Matching and similarity threshold $s^0 = 0.99$.

**Rogers-Tanimoto $s^0 = 0.85$ set**	**nb.clusters**		**p-value**
**patil**			
	2	MR pearson average	0.4056
		MR pearson weighted	
		MR sim weighted	
		MR sim average	
	3	MR sim average	0.0609
		STATUS agree average	
		MR agree average	
		STATUS sim average	
		MR pearson average	
		STATUS manhattan average	
		RATIO manhattan average	
		SMOOTH manhattan average	
		STATUS sim diana	
		MR sim weighted	
		RATIO euclidean average	
		SMOOTH euclidean average	
		PCA euclidean average	
	4	PCA euclidean average	0.6660
		SMOOTH euclidean average	
		STATUS euclidean average	
		MR sim diana	

Table S 111. Stable class discovery methods and associated p-value for data set **patil**, partitions from 2 to 4 clusters, similarity coefficient Rogers-Tanimoto and similarity threshold $s^0 = 0.85$.

**Rogers-Tanimoto $s^0 = 0.85$ set**	**nb.clusters**		**p-value**
**patil**			
	5	STATUS agree average	0.0771
		PCA euclidean average	
		MR sim average	
		MR agree average	
		STATUS sim average	
		SMOOTH euclidean average	
		RATIO euclidean average	
		SMOOTH euclidean diana	
		MR sim diana	
		PCA euclidean diana	
	6	RATIO euclidean average	0.4908
		STATUS agree average	
		STATUS sim average	
		MR sim average	
		SMOOTH euclidean average	
		MR agree average	
		RATIO euclidean diana	
		PCA euclidean average	
	7	MR agree average	0.0900
		SMOOTH euclidean average	
		STATUS agree average	
		RATIO euclidean average	
		MR sim average	
		RATIO euclidean diana	
	8	RATIO euclidean average	0.0931
		SMOOTH euclidean average	
		MR agree average	
		MR sim average	
		PCA manhattan average	
		PCA euclidean average	
		SMOOTH euclidean diana	
		STATUS agree average	
	9	RATIO euclidean average	0.0513
		MR agree average	
		MR sim average	
	10	MR agree average	1

Table S 112. Stable class discovery methods and associated p-value for data set **patil**, partitions from 5 to 10 clusters, similarity coefficient Rogers-Tanimoto and similarity threshold $s^0 = 0.85$.

**Rogers-Tanimoto $s^0 = 0.90$ set**	**nb.clusters**		**p-value**
**patil**			
	2	MR pearson average	0.4056
		MR pearson weighted	
		MR sim weighted	
		MR sim average	
	3	MR sim average	0.0609
		STATUS agree average	
		MR agree average	
		STATUS sim average	
		MR pearson average	
		STATUS manhattan average	
		RATIO manhattan average	
		SMOOTH manhattan average	
		STATUS sim diana	
		MR sim weighted	
		RATIO euclidean average	
		SMOOTH euclidean average	
		PCA euclidean average	
	4	PCA euclidean average	0.6660
		SMOOTH euclidean average	
		STATUS euclidean average	
		MR sim diana	

Table S 113. Stable class discovery methods and associated p-value for data set **patil**, partitions from 2 to 4 clusters, similarity coefficient Rogers-Tanimoto and similarity threshold $s^0 = 0.90$.

Rogers-Tanimoto $s^0 = 0.90$ set	nb.clusters		p-value
**patil**			
	5	STATUS agree average	0.0771
		PCA euclidean average	
		MR sim average	
		MR agree average	
		STATUS sim average	
		SMOOTH euclidean average	
		RATIO euclidean average	
		SMOOTH euclidean diana	
		MR sim diana	
		PCA euclidean diana	
	6	RATIO euclidean average	0.4908
		STATUS agree average	
		STATUS sim average	
		MR sim average	
		SMOOTH euclidean average	
		MR agree average	
		RATIO euclidean diana	
		PCA euclidean average	
	7	MR agree average	0.0900
		SMOOTH euclidean average	
		STATUS agree average	
		RATIO euclidean average	
		MR sim average	
		RATIO euclidean diana	
	8	RATIO euclidean average	0.0931
		SMOOTH euclidean average	
		MR agree average	
		MR sim average	
		PCA manhattan average	
		PCA euclidean average	
		SMOOTH euclidean diana	
		STATUS agree average	
	9	RATIO euclidean average	0.0513
		MR agree average	
		MR sim average	
	10	MR agree average	1

Table S 114. Stable class discovery methods and associated p-value for data set **patil**, partitions from 5 to 10 clusters, similarity coefficient Rogers-Tanimoto and similarity threshold $s^0 = 0.90$.

**Rogers-Tanimoto** $s^0 = 0.95$ **set**	**nb.clusters**		**p-value**
**patil**			
	2	MR pearson average	0.4056
		MR pearson weighted	
		MR sim weighted	
		MR sim average	
	3	MR sim average	0.0609
		STATUS agree average	
		MR agree average	
		STATUS sim average	
		MR pearson average	
		STATUS manhattan average	
		RATIO manhattan average	
		SMOOTH manhattan average	
		STATUS sim diana	
		MR sim weighted	
		RATIO euclidean average	
		SMOOTH euclidean average	
		PCA euclidean average	
	4	PCA euclidean average	0.6660
		SMOOTH euclidean average	
		STATUS euclidean average	
		MR sim diana	

Table S 115. Stable class discovery methods and associated p-value for data set **patil**, partitions from 2 to 4 clusters, similarity coefficient Rogers-Tanimoto and similarity threshold $s^0 = 0.95$.

Rogers-Tanimoto $s^0 = 0.95$ set	nb.clusters		p-value
**patil**			
	5	STATUS agree average	0.0771
		PCA euclidean average	
		MR sim average	
		MR agree average	
		STATUS sim average	
		SMOOTH euclidean average	
		RATIO euclidean average	
		SMOOTH euclidean diana	
		MR sim diana	
		PCA euclidean diana	
	6	RATIO euclidean average	0.4908
		STATUS agree average	
		STATUS sim average	
		MR sim average	
		SMOOTH euclidean average	
		MR agree average	
		RATIO euclidean diana	
		PCA euclidean average	
	7	MR agree average	0.0900
		SMOOTH euclidean average	
		STATUS agree average	
		RATIO euclidean average	
		MR sim average	
		RATIO euclidean diana	
	8	RATIO euclidean average	0.0931
		SMOOTH euclidean average	
		MR agree average	
		MR sim average	
		PCA manhattan average	
		PCA euclidean average	
		SMOOTH euclidean diana	
		STATUS agree average	
	9	RATIO euclidean average	0.0513
		MR agree average	
		MR sim average	
	10	MR agree average	1

Table S 116. Stable class discovery methods and associated p-value for data set **patil**, partitions from 5 to 10 clusters, similarity coefficient Rogers-Tanimoto and similarity threshold $s^0 = 0.95$.

Rogers-Tanimoto $s^0 = 0.97$ set	nb.clusters		p-value
**patil**			
	2	MR pearson average	0.4056
		MR pearson weighted	
		MR sim weighted	
		MR sim average	
	3	MR sim average	0.0609
		STATUS agree average	
		MR agree average	
		STATUS sim average	
		MR pearson average	
		STATUS manhattan average	
		RATIO manhattan average	
		SMOOTH manhattan average	
		STATUS sim diana	
		MR sim weighted	
		RATIO euclidean average	
		SMOOTH euclidean average	
		PCA euclidean average	
	4	PCA euclidean average	0.6660
		SMOOTH euclidean average	
		STATUS euclidean average	
		MR sim diana	

Table S 117. Stable class discovery methods and associated p-value for data set **patil**, partitions from 2 to 4 clusters, similarity coefficient Rogers-Tanimoto and similarity threshold $s^0 = 0.97$.

Rogers-Tanimoto $s^0 = 0.97$ set	nb.clusters		p-value
**patil**			
	5	STATUS agree average	0.0771
		PCA euclidean average	
		MR sim average	
		MR agree average	
		STATUS sim average	
		SMOOTH euclidean average	
		RATIO euclidean average	
		SMOOTH euclidean diana	
		MR sim diana	
		PCA euclidean diana	
	6	RATIO euclidean average	0.4908
		STATUS agree average	
		STATUS sim average	
		MR sim average	
		SMOOTH euclidean average	
		MR agree average	
		RATIO euclidean diana	
		PCA euclidean average	
	7	MR agree average	0.0900
		SMOOTH euclidean average	
		STATUS agree average	
		RATIO euclidean average	
		MR sim average	
		RATIO euclidean diana	
	8	RATIO euclidean average	0.0931
		SMOOTH euclidean average	
		MR agree average	
		MR sim average	
		PCA manhattan average	
		PCA euclidean average	
		SMOOTH euclidean diana	
		STATUS agree average	
	9	RATIO euclidean average	0.0513
		MR agree average	
		MR sim average	
	10	MR agree average	1

Table S 118. Stable class discovery methods and associated p-value for data set **patil**, partitions from 5 to 10 clusters, similarity coefficient Rogers-Tanimoto and similarity threshold $s^0 = 0.97$.

**Rogers-Tanimoto** $s^0 = 0.99$ **set**	**nb.clusters**		**p-value**
**patil**			
	2	MR pearson average	0.4056
		MR pearson weighted	
		MR sim weighted	
		MR sim average	
	3	MR sim average	0.0609
		STATUS agree average	
		MR agree average	
		STATUS sim average	
		MR pearson average	
		STATUS manhattan average	
		RATIO manhattan average	
		SMOOTH manhattan average	
		STATUS sim diana	
		MR sim weighted	
		RATIO euclidean average	
		SMOOTH euclidean average	
		PCA euclidean average	
	4	PCA euclidean average	0.6660
		SMOOTH euclidean average	
		STATUS euclidean average	
		MR sim diana	

Table S 119. Stable class discovery methods and associated p-value for data set **patil**, partitions from 2 to 4 clusters, similarity coefficient Rogers-Tanimoto and similarity threshold $s^0 = 0.99$.

**Rogers-Tanimoto** $s^0 = 0.99$ **set**	**nb.clusters**		**p-value**
**patil**			
	5	STATUS agree average	0.0771
		PCA euclidean average	
		MR sim average	
		MR agree average	
		STATUS sim average	
		SMOOTH euclidean average	
		RATIO euclidean average	
		SMOOTH euclidean diana	
		MR sim diana	
		PCA euclidean diana	
	6	RATIO euclidean average	0.4908
		STATUS agree average	
		STATUS sim average	
		MR sim average	
		SMOOTH euclidean average	
		MR agree average	
		RATIO euclidean diana	
		PCA euclidean average	
	7	MR agree average	0.0900
		SMOOTH euclidean average	
		STATUS agree average	
		RATIO euclidean average	
		MR sim average	
		RATIO euclidean diana	
	8	RATIO euclidean average	0.0931
		SMOOTH euclidean average	
		MR agree average	
		MR sim average	
		PCA manhattan average	
		PCA euclidean average	
		SMOOTH euclidean diana	
		STATUS agree average	
	9	RATIO euclidean average	0.0513
		MR agree average	
		MR sim average	
	10	MR agree average	1

Table S 120. Stable class discovery methods and associated p-value for data set **patil**, partitions from 5 to 10 clusters, similarity coefficient Rogers-Tanimoto and similarity threshold $s^0 = 0.99$.

nb of clusters	class discovery method	χ^2-based test p.value	empirical similarity mean with Jaccard coefficient	empirical similarity variance with Jaccard coefficient	χ^2-based test corrected p.value
2	MR agree average	1	0.9853687	2.635496e-05	1
3	MR agree average	1	0.9710836	5.662734e-05	1
4	MR agree average	1	0.9569568	9.837115e-05	1
	Calls sim ward	0.1742314	0.7650508	1.470865e-02	0.3484628
5	MR agree average	1	0.9441099	0.0001196906	1
6	MR agree average	1	0.9316517	0.0001279881	1

Table S 121. Class discovery methods declared stable for data set **zhang**.

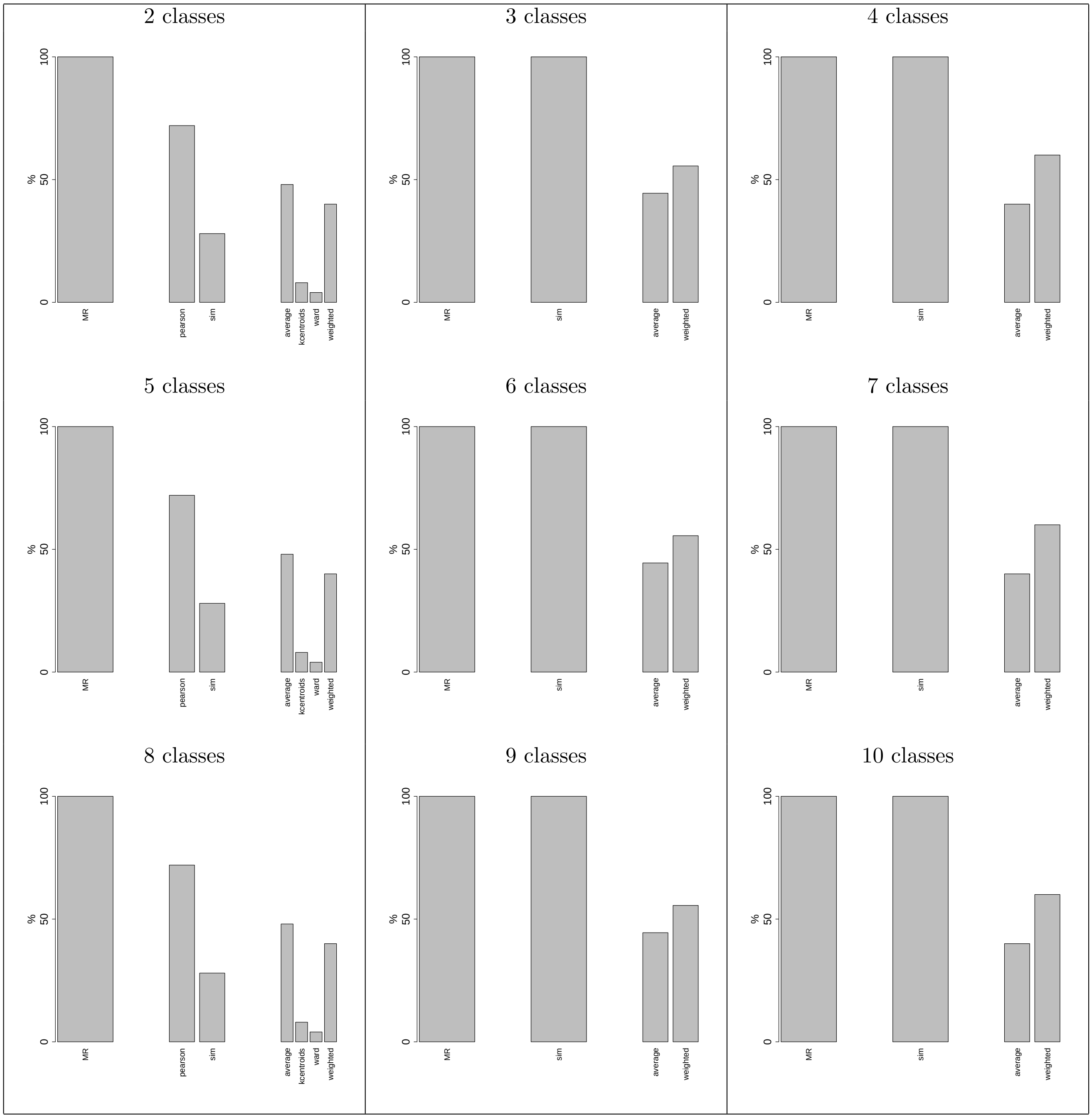

Figure S 2. Frequency of input data representation, dissimilarity measure and clustering algorithm among the class discovery methods declared stable for each partition from 2 to 10 clusters for the data set **blaveri**. The parameters used are Jaccard coefficient and 0.97 threshold.

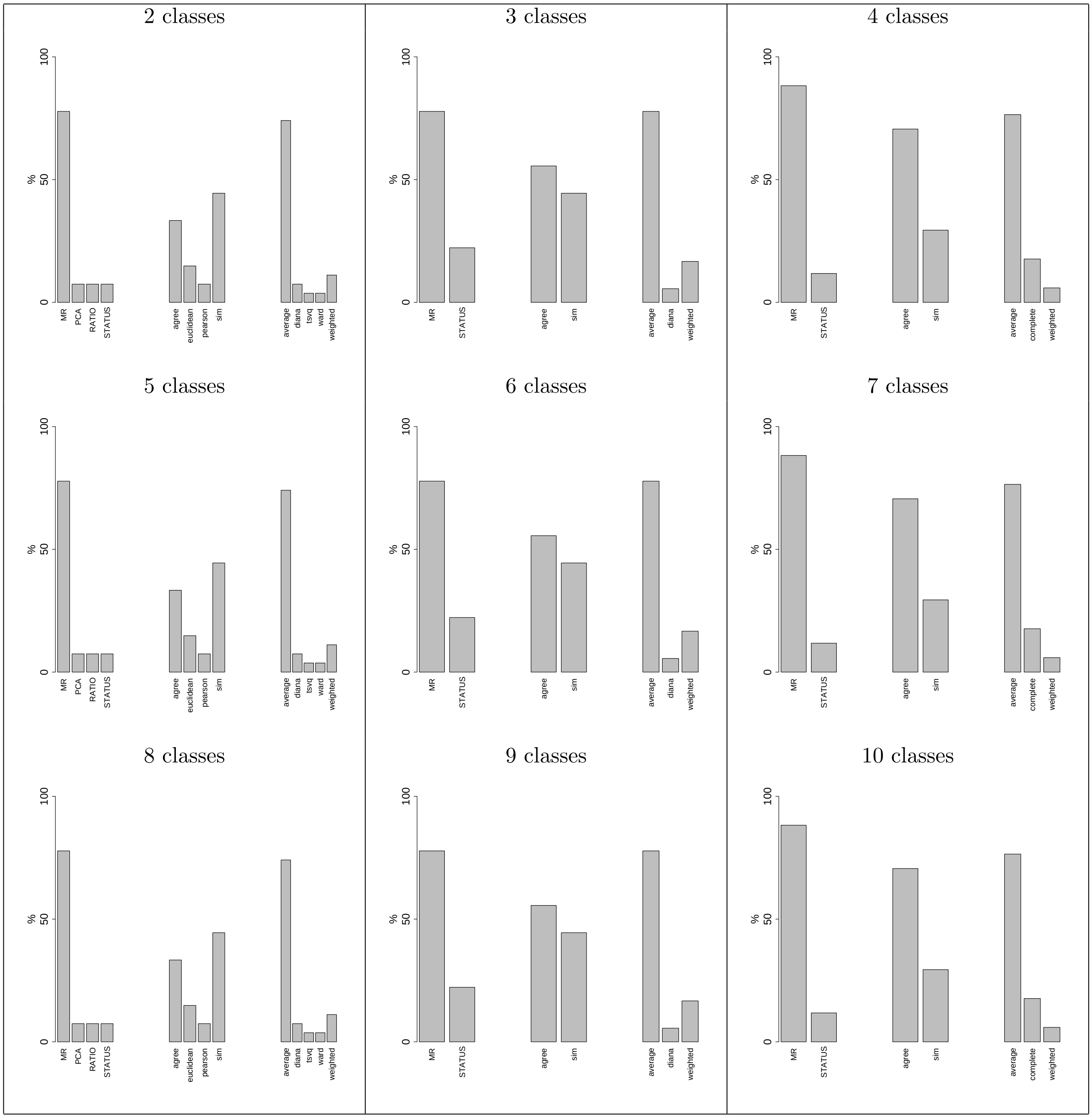

Figure S 3. Frequency of input data representation, dissimilarity measure and clustering algorithm among the class discovery methods declared stable for each partition from 2 to 10 clusters for the data set **gysin**. The parameters used are Jaccard coefficient and 0.97 threshold.

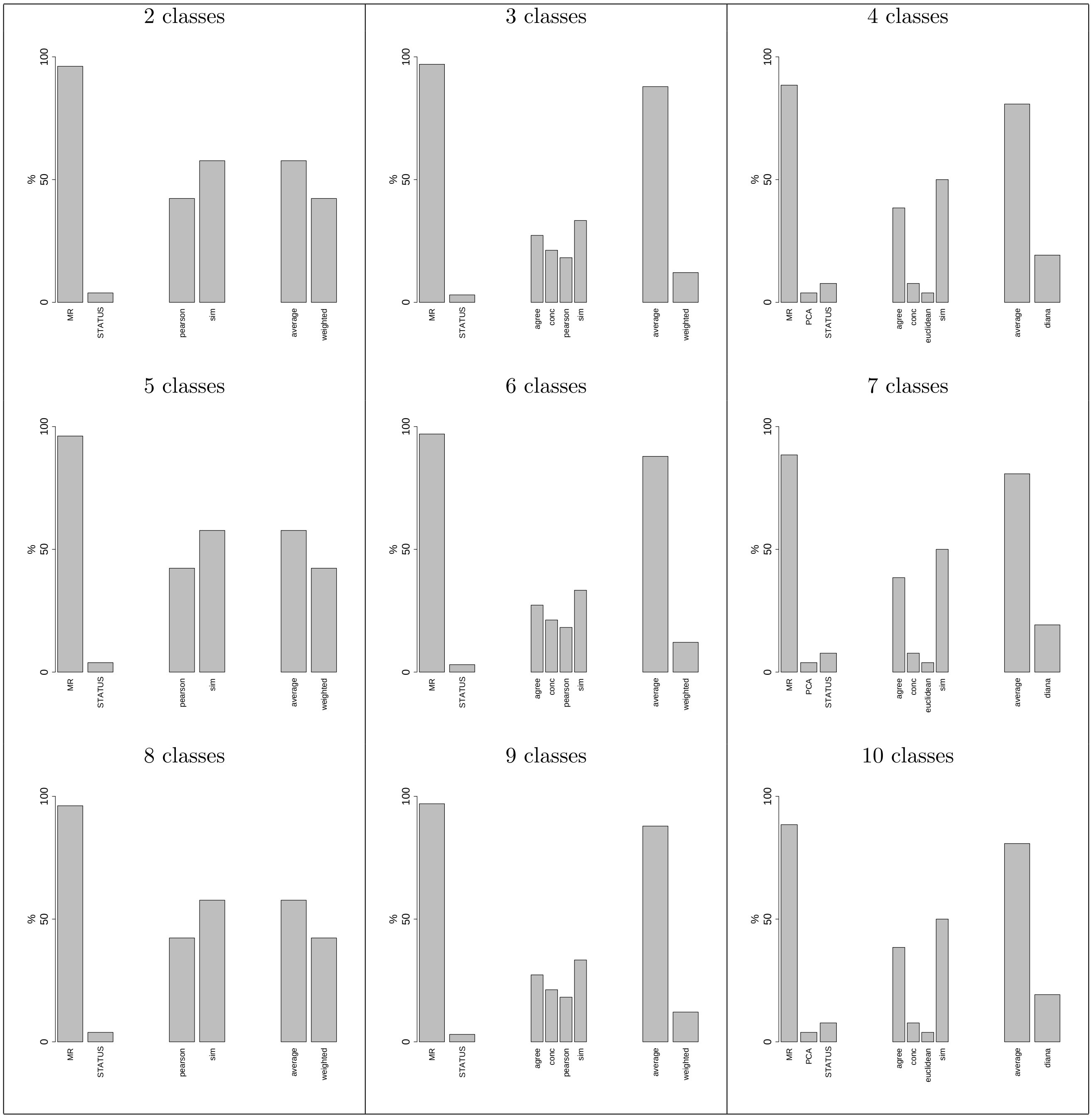

Figure S 4. Frequency of input data representation, dissimilarity measure and clustering algorithm among the class discovery methods declared stable for each partition from 2 to 10 clusters for the data set **douglas**. The parameters used are Jaccard coefficient and 0.97 threshold.

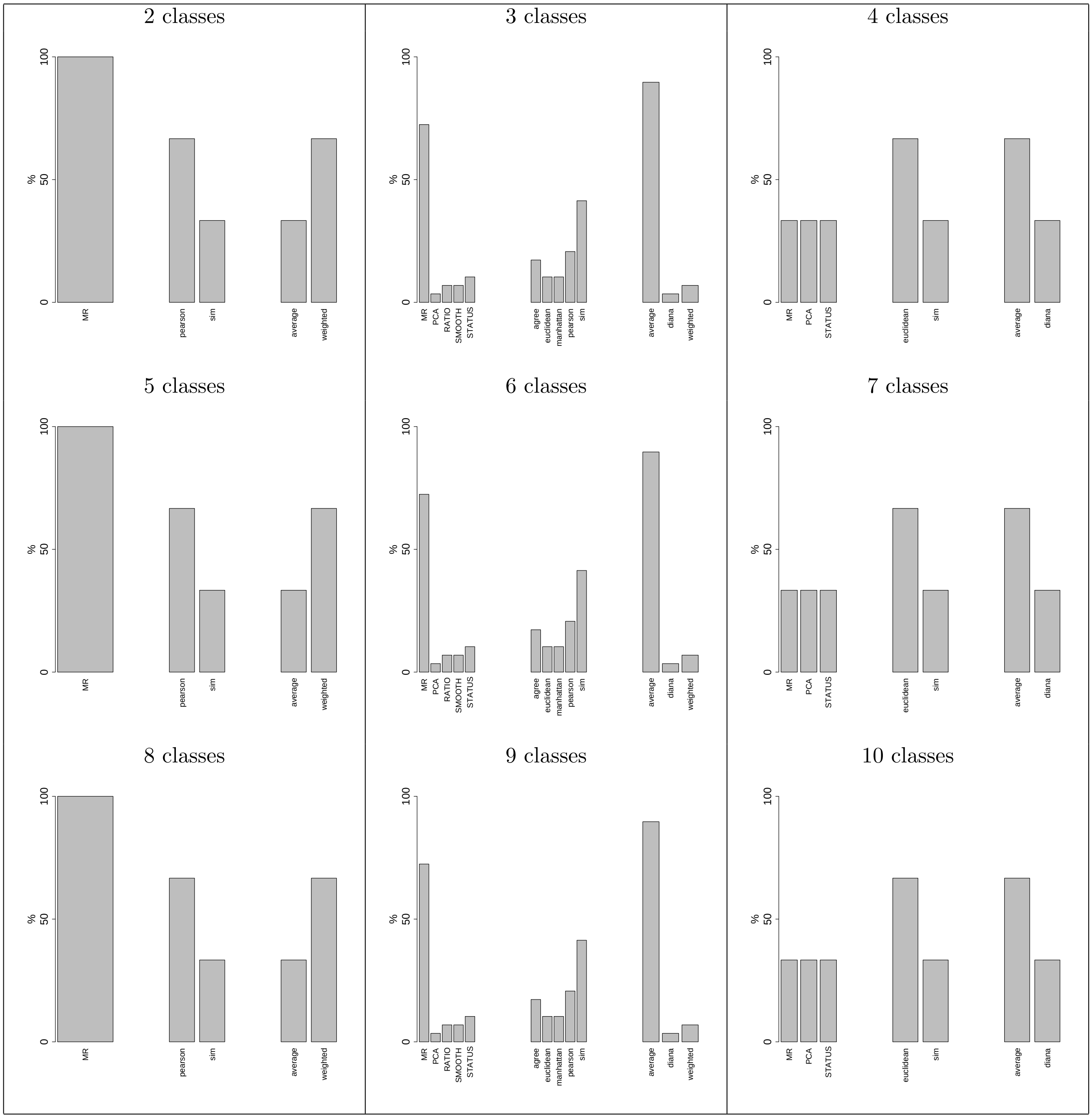

Figure S 5. Frequency of input data representation, dissimilarity measure and clustering algorithm among the class discovery methods declared stable for each partition from 2 to 10 clusters for the data set **patil**. The parameters used are Jaccard coefficient and 0.97 threshold.

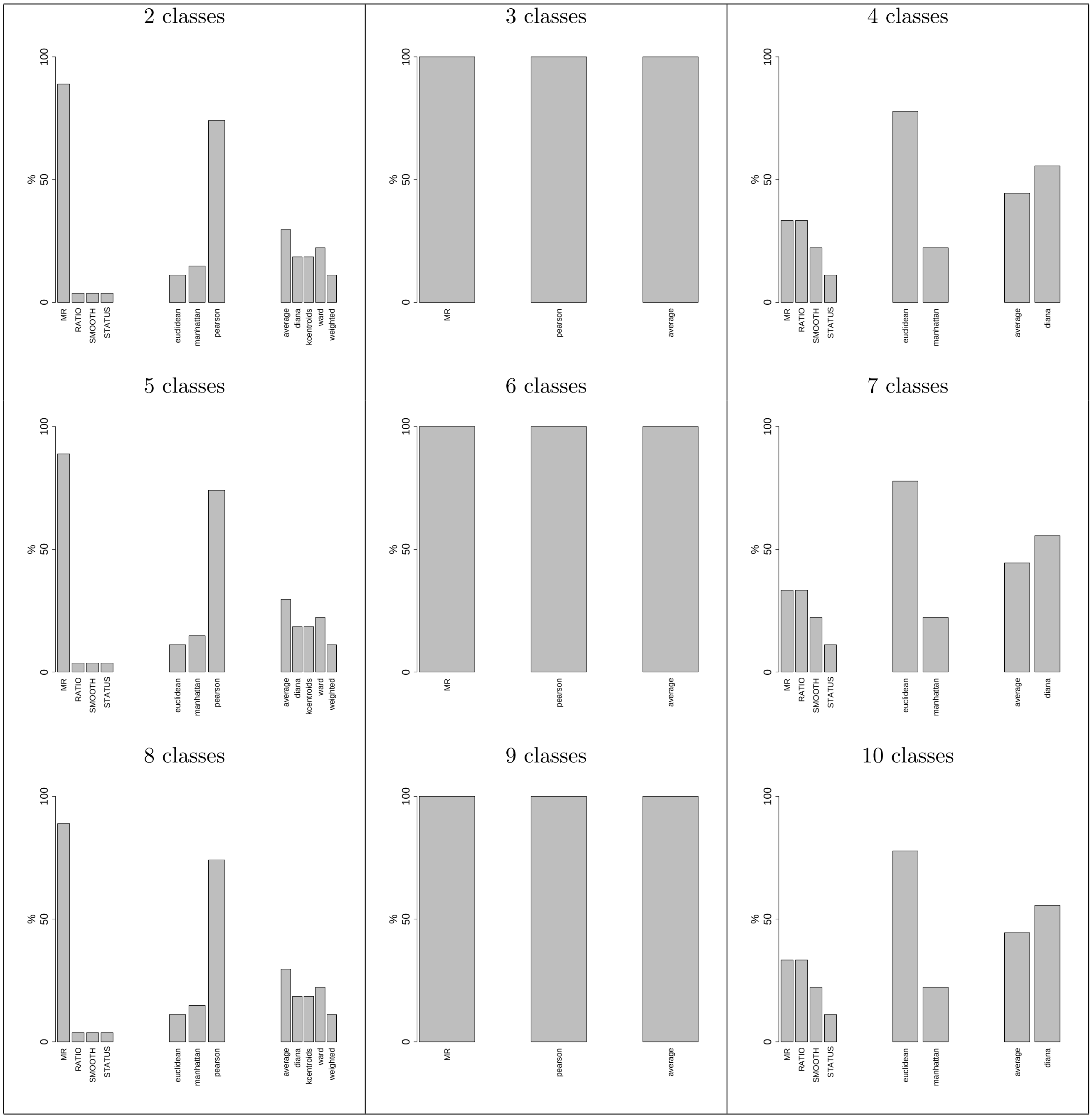

Figure S 6. Frequency of input data representation, dissimilarity measure and clustering algorithm among the class discovery methods declared stable for each partition from 2 to 10 clusters for the data set **veltman**. The parameters used are Jaccard coefficient and 0.97 threshold.

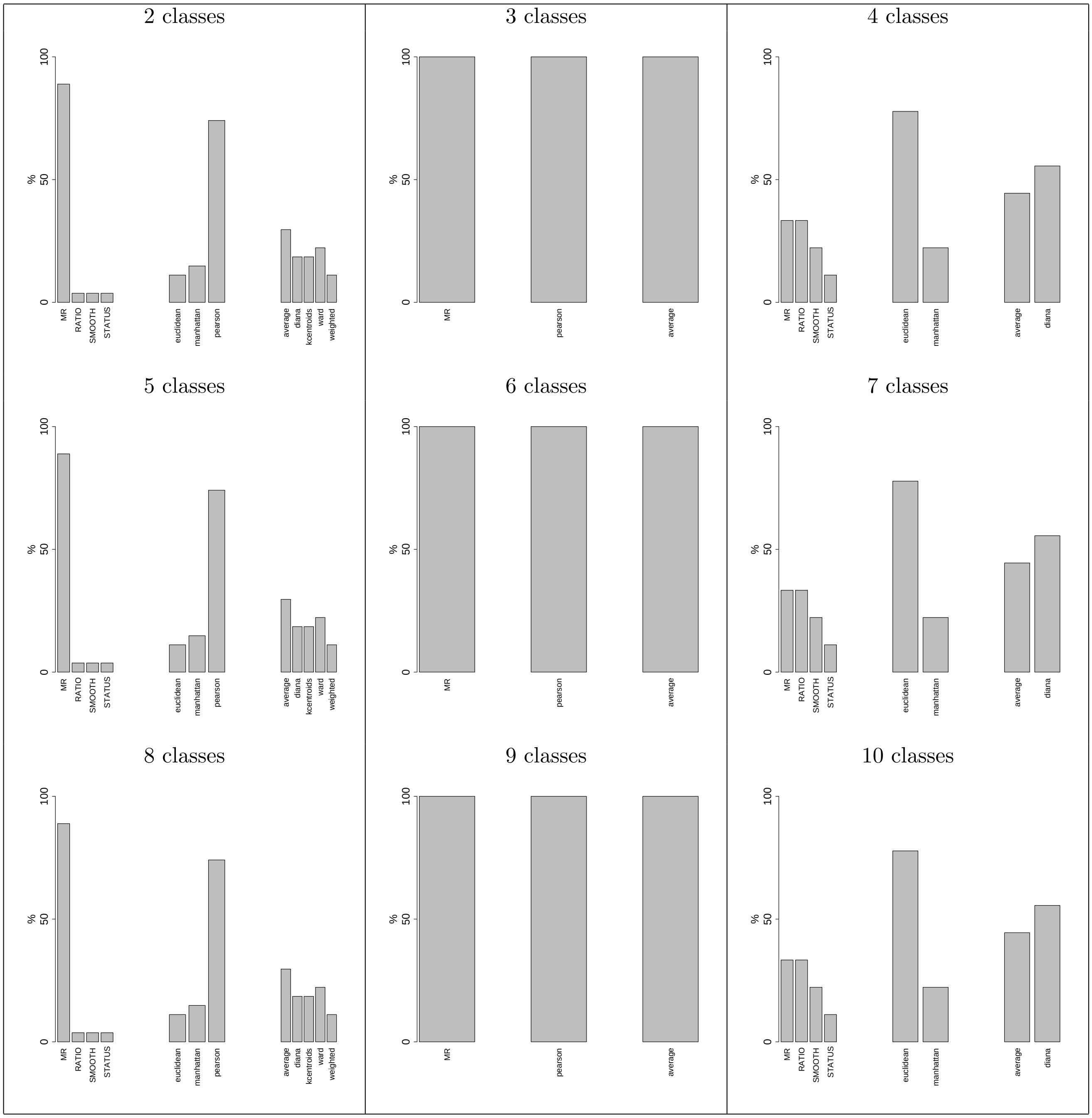

Figure S 7. Frequency of input data representation, dissimilarity measure and clustering algorithm among the class discovery methods declared stable for each partition from 2 to 10 clusters for the data set **veltman**. The parameters used are Jaccard coefficient and 0.97 threshold.

Bibliography

[1] Kaufman, L. and Rousseeuw, P., Finding groups in data, Wiley, 1990, New York.

[2] Macnaughton-Smith, P. and Williams, W. and Dale, M. and Mockett, L., Dissimilarity analysis: a new technique of hierarchical sub-division, Nature, 1964, 202, 1034-1035.

[3] Chipman, H. and Tibshirani, R., Hybrid hierarchical clustering with applications to microarray data, Biostatistics, 2006, 7, 2, 286-301.

[4] Gersho, A. and Gray, R., Vector Quantization and Signal Compression, Kluwer Academic, 1992, Boston.

[5] Brock, G., Shaffer, J., Blakesley, R., Lotz, M., and Tseng, G., Which missing value imputation method to use in expression profiles: a comparative study and two selection schemes, BMC Bioinformatics, 2008, 10, 912.

[6] Cha, S-H., Tappert, C., and Yoon, S., Enhancing Binary Feature Vector Similarity Measures, Journal of Pattern Recognition Research, 2006, 1, 63-77.
